# Mouse models for hereditary spastic paraplegia uncover a role of PI4K2A in autophagic lysosome reformation

**DOI:** 10.1080/15548627.2021.1891848

**Published:** 2021-03-09

**Authors:** Mukhran Khundadze, Federico Ribaudo, Adeela Hussain, Henry Stahlberg, Nahal Brocke-Ahmadinejad, Patricia Franzka, Rita-Eva Varga, Milena Zarkovic, Thanakorn Pungsrinont, Miriam Kokal, Ian G. Ganley, Christian Beetz, Marc Sylvester, Christian A. Hübner

**Affiliations:** a Institute of Human Genetics, University Hospital Jena, Friedrich-Schiller-University Jena, Jena, Germany; b Core Facility Mass Spectrometry, Institute of Biochemistry and Molecular Biology, Medical Faculty, University of Bonn, Bonn, Germany; c MRC Protein Phosphorylation and Ubiquitylation Unit, School of Life Sciences, University of Dundee, Dundee, Scotland; d Institute of Clinical Chemistry, University Hospital Jena, Friedrich-Schiller-University Jena, Germany; Current Affiliation: Centogene GmbH, Rostock, Germany

**Keywords:** Autophagy, lysosome, neurodegeneration, PI4K2A, spg11, spg15

## Abstract

Hereditary spastic paraplegia (HSP) denotes genetically heterogeneous disorders characterized by leg spasticity due to degeneration of corticospinal axons. SPG11 and SPG15 have a similar clinical course and together are the most prevalent autosomal recessive HSP entity. The respective proteins play a role for macroautophagy/autophagy and autophagic lysosome reformation (ALR). Here, we report that *spg11* and *zfyve26* KO mice developed motor impairments within the same course of time. This correlated with enhanced accumulation of autofluorescent material in neurons and progressive neuron loss. In agreement with defective ALR, tubulation events were diminished in starved KO mouse embryonic fibroblasts (MEFs) and lysosomes decreased in neurons of KO brain sections. Confirming that both proteins act in the same molecular pathway, the pathologies were not aggravated upon simultaneous disruption of both. We further show that PI4K2A (phosphatidylinositol 4-kinase type 2 alpha), which phosphorylates phosphatidylinositol to phosphatidylinositol-4-phosphate (PtdIns4P), accumulated in autofluorescent deposits isolated from KO but not WT brains. Elevated PI4K2A abundance was already found at autolysosomes of neurons of presymptomatic KO mice. Immunolabelings further suggested higher levels of PtdIns4P at LAMP1-positive structures in starved KO MEFs. An increased association with LAMP1-positive structures was also observed for clathrin and DNM2/dynamin 2, which are important effectors of ALR recruited by phospholipids. Because PI4K2A overexpression impaired ALR, while its knockdown increased tubulation, we conclude that PI4K2A modulates phosphoinositide levels at autolysosomes and thus the recruitment of downstream effectors of ALR. Therefore, PI4K2A may play an important role in the pathogenesis of SPG11 and SPG15.

**Abbreviations**: ALR: autophagic lysosome reformation; AP-5: adaptor protein complex 5; BFP: blue fluorescent protein; dKO: double knockout; EBSS: Earle’s balanced salt solution; FBA: foot base angle; GFP: green fluorescent protein; HSP: hereditary spastic paraplegia; KO: knockout; LAMP1: lysosomal-associated membrane protein 1; MAP1LC3B/LC3: microtubule-associated protein 1 light chain 3 beta; MEF: mouse embryonic fibroblast; SQSTM1/p62: sequestosome 1; PI4K2A: phosphatidylinositol 4-kinase type 2 alpha; PtdIns3P: phosphatidylinositol-3-phosphate; PtdIns4P: phosphatidylinositol-4-phosphate; RFP: red fluorescent protein; SPG: spastic paraplegia gene; TGN: trans-Golgi network; WT: wild type

## Introduction

Hereditary spastic paraplegia (HSP) refers to a group of neurodegenerative disorders, which are characterized by the degeneration of upper motoneuron axons thus resulting in lower limb spasticity and weakness [1]. Additional symptoms such as cognitive deficits, a thinning of the corpus callosum, optic atrophy, cerebellar atrophy, amyotrophy, peripheral nerve involvement, and seizures can occur and characterize complicated HSPs. Up to date more than 80 different genetic loci have been identified, which are termed as SPGs with a running number reflecting the order of its identification. Mutations in SPG11 (SPG11 vesicle trafficking associated, spatacsin) are the most common cause of autosomal recessively inherited HSP [2]. Affected patients often suffer from mental retardation, ataxia, Parkinsonism, retinopathy or polyneuropathy and show a thinning of the corpus callosum and cortical atrophy. Clinically SPG11 patients cannot be distinguished from SPG15 patients, another autosomal recessive form of HSP [3], which is caused by mutations in ZFYVE26/Spastizin (zinc finger FYVE-type containing 26). Therefore, it was concluded that SPG11 and ZFYVE26 may act in the same cellular pathway. Indeed, both SPG11 and ZFYVE26 co-precipitate with other proteins [4], which were later identified as members of the newly identified adaptor protein (AP) complex AP-5 [5].

AP complexes mediate intracellular membrane trafficking along endocytic and secretory transport pathways. It is assumed that AP-5 serves as a backup system for the retrieval of proteins from late endosomes to the *trans* Golgi, because knockdown [6] or disruption [7] of its AP5Z1/ζ-subunit impair the retrieval of some Golgi-related proteins from endosomes back to the Golgi. Notably, mutations in AP5Z1 cause SPG48 [4], which shows many similarities with SPG11 and SPG15 [8]. Because SPG11 and ZFYVE26 have predicted secondary structures containing alpha-solenoids related to those of clathrin heavy chain and COPI (coatomer protein complex I) subunits and because SPG11 also has an N-terminal, beta-propeller-like domain, which interacts with AP-5, both may contribute to the scaffold of AP-5 [9]. Thus, AP-5 might be involved in protein sorting, while ZFYVE26 facilitates the docking of the coat onto membranes by interacting with PtdIns3P via its FYVE domain and SPG11 forms the scaffold.

Because AP-5 subunits have been coordinately lost in many organisms, in which the SPG11 and ZFYVE26 orthologs are still present, it was proposed that ZFYVE26 and SPG11 may also serve functions independent of AP-5 [5]. A role in cell division [10], DNA repair [4], endosomal trafficking [9], autophagy [11] and autophagic lysosome reformation (ALR) [12–14] has been reported. The latter mechanism is regulated by MTOR (mechanistic target of rapamycin kinase), which allows the recycling of lysosomes from autolysosomes after prolonged starvation [15]. It was proposed that defective ALR may alter the subcellular distribution of cholesterol and thus affect intracellular calcium homeostasis, which may impair lysosome tubulation via TFEB (transcription factor EB), the master regulator of lysosomes [16].

We and others characterized knockout (KO) mouse models for either SPG11 [14,17] or ZFYVE26 [7] and AP5Z1 [18]. Both *spg11* KO and *zfyve26* KO mice developed a gait disorder compatible with HSP. As a correlate we found a progressive intracellular accumulation of LAMP1- and SQSTM1-positive autofluorescent material in cortical motoneurons and in Purkinje neurons of *spg11* KO and *zfyve26* KO mice, which are finally lost. Supporting a defect of autophagy, levels of lipidated LC3 were increased in *spg11* KO mouse embryonic fibroblasts (MEFs). In agreement with a possible defect of ALR, lysosome numbers were reduced in *spg11* KO Purkinje neurons *in vivo*.

Here, we report the generation of *spg11 zfyve26* double KO (dKO) mice. We observed a similar time dependent massive accumulation of lysosome-related autofluorescent material in neurons, progressive neuron loss and motor impairments in KO and dKO mice adding support to the notion that SPG11 and ZFYVE26 act together. Our unbiased mass spectrometry approach identified PI4K2A (phosphatidylinositol 4-kinase type 2 alpha) in autofluorescent material isolated from brains of *spg11* KO and *zfyve26* KO but not WT mice. Immunostainings of brain sections further revealed that PI4K2A abundance is already increased at autolysosomes of KO mice before onset of overt neurodegeneration. PI4K2A overexpression in U2-OS cells inhibited autolysosomal tubulation and increased the association of clathrin and DNM2/dynamin 2 with LAMP1-positive structures similar to our findings in KO MEFs. Here, we propose that increased PI4K2A abundance contributes to the pathophysiology of SPG11 and SPG15, because PI4K2A modulates phosphoinositide levels at autolysosomes and thus down-stream effectors involved in ALR.

## Results

### The simultaneous disruption of *Spg11* in *zfyve26* KO mice does not aggravate the phenotype of *spg11* KO or *zfyve26* KO mice

**Figure 1.**
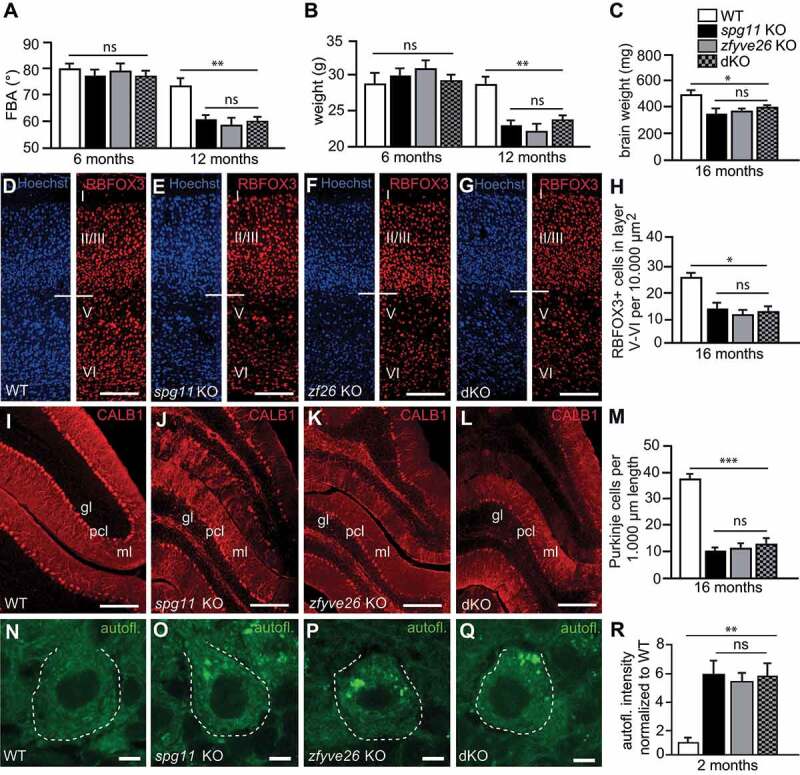
*Spg11* KO and *zfyve26* KO mice share the same phenotype which is not aggravated by simultaneous disruption of both. (A) At 6 months of age the mean foot base angle (FBA) at toe-off position is not altered in *spg11* KO, *zfyve26* KO, and *spg11 zfyve26* double KO (dKO) compared to WT mice. Six months later, the FBA is likewise decreased in *spg11* KO, *zfyve26* KO and dKO mice compared to WT (n = 6 mice per genotype; two-way ANOVA followed by Bonferroni test; ** p < 0.01; ns: not significant). (B) The mean body weight of *spg11* KO, *zfyve26* KO and dKO compared to control mice is decreased at 12 months of age (n = 6 mice per genotype for body weight; two-way ANOVA followed by Bonferroni test; * p < 0.05; ns: not significant). (C) The mean brain weight is significantly reduced in 16-months-old KO and dKO mice (n = 4 mice per genotype for brain weight; one-way ANOVA followed by Tukey’s Multiple Comparison Test; * p < 0.05; ns: not significant). (D-H) Compared to WT (D) RBFOX3 stained neurons (red) in motor cortex layers V–VI of 16-months-old *spg11* KO (E), *zfyve26* KO (F) and dKO (G) mice are likewise reduced (H; n = 3 mice per genotype with n = 1 section; one-way ANOVA followed by Tukey’s Multiple Comparison Test; * indicates p < 0.05; ns: not significant). Individual cortical layers are labeled (I–VI). Scale bars: 100 μm. (I-M) Compared to WT (I) mice, CALB1 labeled Purkinje cells (red) are decreased as well in 16-months-old *spg11* KO (J), *zfyve26* KO (K) and dKO (L) mice (n = 3 mice per genotype with n = 1 section; one-way ANOVA followed by Tukey’s Multiple Comparison Test; *** indicates p < 0.001; ns: not significant). ml: molecular layer; pcl: Purkinje cell layer; gl: granular layer. Scale bars: 100 μm. (N-R) Compared to WT (N) autofluorescence (green, excitation at 488 nm) is similarly increased in Purkinje cells of 2-months-old *spg11* KO (O), *zfyve26* KO (P) and dKO (Q) mice (at least 10 cells per mouse; n = 3 mice per genotype; one-way ANOVA followed by Tukey’s Multiple Comparison Test; ** indicates p < 0.01; ns: not significant). Scale bars: 5 μm. Error bars represent SEM

We previously generated *zfyve26* KO [7] and *spg11* KO [14] mouse models. Since the initial description both lines had been backcrossed with C57BL/6 J for more than 10 generations resulting in an almost pure C57BL/6 J background. To get more insights into the molecular function of SPG11 or ZFYVE26 we crossed both lines and obtained mice heterozygous for both the *spg11* KO and the *zfyve26* KO allele. Notably, double heterozygous KO mice did not develop any gait abnormalities (data not shown), even beyond 18 months of age. Mating of double heterozygous KO mice finally resulted in homozygous s*pg11 zfyve26* double knockout (dKO) mice, which were born in the expected Mendelian ratio. To compare the severity of the motor phenotypes of the different genotypes we measured the foot-base-angle (FBA) at toe-off position of the hind-paw over time. While there was no difference between genotypes at 6 months of age, the FBA was similarly flatter in *spg11* KO, *zfyve26* KO and dKO mice at 12 months of age as compared to WT (wild-type) littermates (Figure 1A). At the same age, we noted a comparable body weight loss, in *spg11* KO, *zfyve26* KO and dKO littermates (Figure 1B). We also measured the brain weight at 16 months of age, which was reduced in *spg11* KO, *zfyve26* KO, and dKO mice compared to WT. Again, there was no difference between single KO and dKO mice (Figure 1C). We further quantified cortical neurons in brain sections stained for the neuronal marker RBFOX3/NeuN (Figure 1D**-G**). Neuron numbers in layer V to VI were reduced in 16-months-old *spg11* KO and *zfyve26* KO compared to WT mice, but the loss was not worsened by simultaneous disruption of *Spg11* and *Zfyve26* (Figure 1H). Similar results were obtained for the loss of Purkinje cells, which were identified by the expression of CALB1/calbindin 1 (Figure 1I**-M**).

Taken together, these data suggest that the phenotype is not aggravated by simultaneous disruption of both *Spg11* and *Zfyve26*.

### Autophagy related autofluorescent material is similarly increased in neurons of *spg11* KO, *zfyve26* KO, and dKO mice

We previously reported that the accumulation of lipofuscin-like autofluorescent intracellular material (emission wavelength between 460 and 630 nm) is drastically increased in Purkinje cells and cortical motoneurons of both *spg11* KO and *zfyve26* KO mice starting from 2 months of age onwards [7,14]. Notably, the autofluorescent material detected in Purkinje cells of mice heterozygous for one *spg11* KO and one *zfyve26* KO allele did not differ from control mice **(Fig. S1A-C**). Autofluorescence quantification for Purkinje cells did not suggest that the intracellular pathology was more severe in dKO mice at 2 (Figure 1N**-R**) or 16 (**Fig. S1D-H**) months of age compared to *spg11* KO and *zfyve26* KO mice.

**Figure 2.**
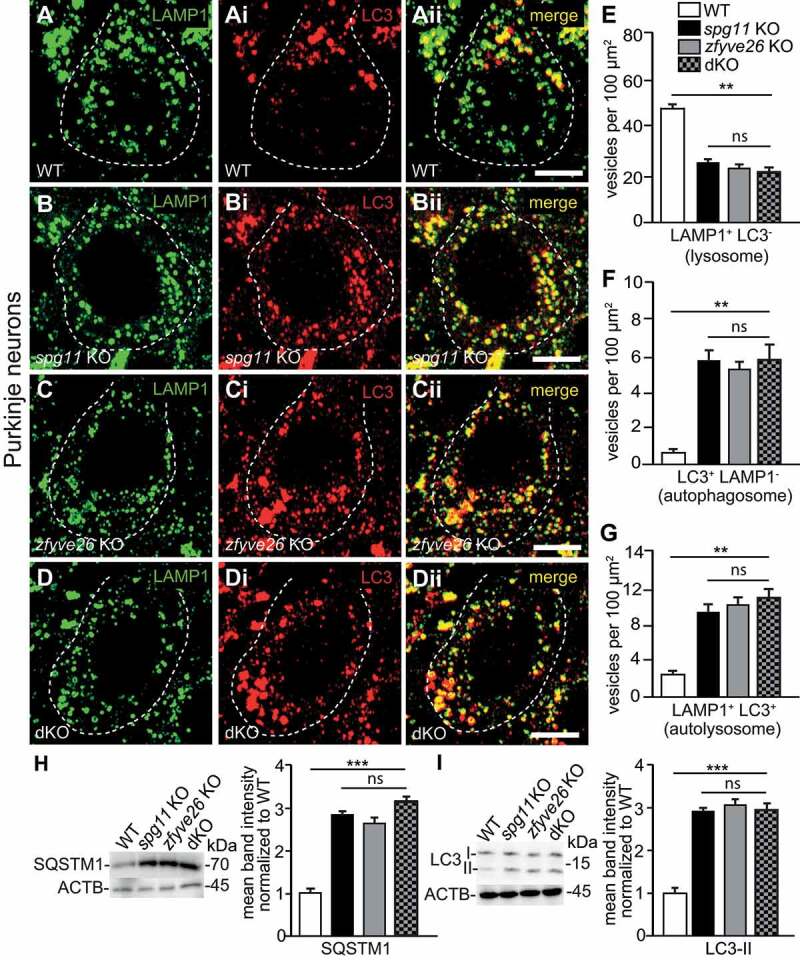
Less lysosomes in Purkinje cells of *spg11* KO, *zfyve26* KO and *spg11 zfyve26* double KO (dKO) mice. (A-E) Lysosomes numbers are likewise reduced in Purkinje cells of 2-months-old *spg11* KO, *zfyve26* KO and dKO mice. Representative Purkinje cells in brain sections of WT (A-Aii), *spg11* KO (B-Bii), *zfyve26* KO (C-Cii) and dKO mice (D-Dii) stained for LAMP1 (green) and LC3 (red). Purkinje cell somata are marked by a dashed line. Quantification of lysosomes defined as LAMP1-positive and LC3-negative puncta (E), autophagosomes defined as LAMP1-negative but LC3-psotive puncta (F) and autolysosomes defined as LAMP1- and LC3-positive puncta (G). More than 30 cells from 3 mice per genotype were analyzed (one-way ANOVA followed by Tukey’s Multiple Comparison Test; ** p < 0.01; ns: not significant). Scale bars: 5 µm. (H and I) SQSTM1 (H) and LC3-II (I) protein abundances were likewise increased in brain lysates of 16-months-old *spg11* KO, *zfyve26* KO and dKO mice. ACTB/β-actin served as loading control (4 mice per genotype; one-way ANOVA followed by Tukey’s Multiple Comparison Test; *** p < 0.001; ns: not significant). Error bars represent SEM

To better characterize the material, we stained brain sections with different markers such as LAMP1, SQSTM1 and LC3. We applied a spectral analysis to distinguish the labeled compartment from autofluorescence using a linear unmixing algorithm as described previously [7]. As already observed in *spg11* KO mice [14], the autofluorescent material in *zfyve26* KO and dKO mice labeled for SQSTM1 (**Fig. S1Di,Ei** and **Fi**) and signal intensities did not differ between genotypes at 16 months of age (**Fig. S1I**). We also co-stained brain sections from 2-months-old WT, KO, and dKO mice for the lysosomal marker LAMP1 and LC3 (Figure 2A**-Dii**), which labels autophagic vesicles. Lysosomes defined as LAMP1-positive but LC3-negative puncta were decreased in KO and dKO mice (Figure 2E), while autophagosomes (LAMP1-negative but LC3-positive puncta, Figure 2F) and autolysosomes (puncta labeled for LAMP1 and LC3, Figure 2G) were increased. Similar results were obtained, when we co-stained brain sections with LAMP1 and SQSTM1 (**Fig. S2**). Again, we did not observe that these alterations were more pronounced in dKO mice.

In accordance with our immunostainings, overall abundances for SQSTM1 and LC3 were increased in brain lysates of 16-months-old *spg11* KO, *zfyve26* KO and dKO mice (Figure 2H and **I**).

Together these findings suggest that the clearance of autophagic material is impaired upon disruption of either *Spg11* or *Zfyve26* and thus accumulates over time.

### Autophagic lysosome reformation is compromised in *spg11* KO, *zfyve26* KO and dKO mice

**Figure 3.**
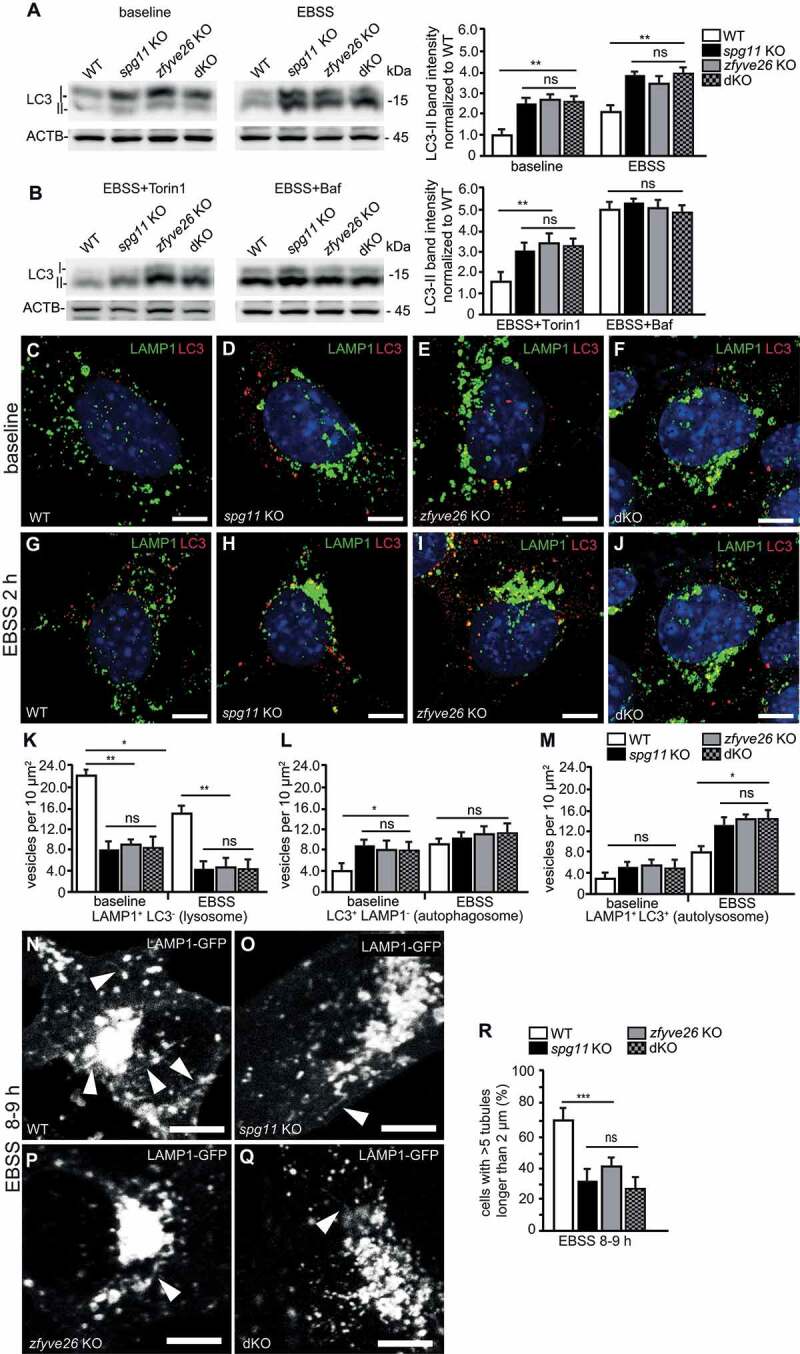
Autophagic lysosome reformation is compromised in *spg11* KO, *zfyve26* KO and *spg11 zfyve26* double KO (dKO) mouse embryonic fibroblasts. (A and B) Defective autophagy in *spg11* KO, *zfyve26* KO and dKO mouse embryonic fibroblasts (MEFs). Semi-quantitative western blotting analysis of LC3 abundance in WT, *spg11* KO, *zfyve26* KO and dKO MEFs at steady state and upon 6 h EBSS starvation with or without Torin1 or bafilomycin A_1_ (Baf). ACTB served as loading control. Normalization is relative to baseline WT (n = 3 experiments; one-way ANOVA followed by Tukey’s Multiple Comparison Test; * p < 0.05; ** p < 0.01; ns: not significant). (C-M) Autophagic flux is similarly compromised in *spg11* KO, *zfyve26* KO and dKO MEFs. LAMP1 (green) and LC3 (red) stainings of WT (C and G), s*pg11* KO (D and H), *zfyve26* KO (E and I) and dKO (F and J) MEFs at baseline and after 2 h of EBSS starvation. Scale bars: 10 µm. (K-M) Quantification of lysosomes (K) defined as LAMP1-positive but LC3-negative puncta, autophagosomes (L) defined as LC3-positive but LAMP1-negative puncta, and autolysosomes (M) defined as LAMP1- and LC3-positive puncta (at least 30 cells per genotype from n = 3 experiments; one-way ANOVA followed by Tukey’s Multiple Comparison Test; ** p < 0.01; * p < 0.05; ns: not significant). (N-R) ALR is similarly compromised in *spg11* KO, *zfyve26* KO and dKO MEFs. Representative live cell imaging time frames of WT (N), *spg11* KO (O), *zfyve26* KO (P) and dKO (Q) MEFs between 8 and 9 h of EBSS starvation. LAMP1-positive tubules are marked by white arrowheads. Scale bars: 10 µm. (R) Quantification of cells with more than 5 LAMP1-positive tubules longer than 2 µm (at least 30 cells per genotype from n = 3 independent experiments; one-way ANOVA followed by Newman-Keuls Multiple Comparison Test; *** p < 0.001; ns: not significant). Error bars represent SEM

To scrutinize the autophagic capacity during starvation, we isolated MEFs from *spg11* KO, *zfyve26* KO and dKO mice. Consistent with a defect of the degradative system, LC3-II abundance was increased in *spg11* KO, *zfyve26* KO, and dKO MEFs at baseline and further increased after starvation with EBSS (Figure 3A). Addition of the mTOR inhibitor Torin 1 during starvation did not increase LC3-II abundance any further. Starvation of cells in the presence of bafilomycin A_1_, which inhibits lysosomal acidification as well as the fusion of lysosomes with autophagosomes [19], abolished the difference in LC3-II abundances between *spg11* KO, *zfyve26* KO, dKO and WT MEFs (Figure 3B). This suggests that the formation of autophagosomes is not impaired upon disruption of *Spg11* or *Zfyve26*.

We co-stained MEFs for LC3 and LAMP1 to distinguish autophagosomes (LC3-positive puncta), lysosomes (LAMP1-positive puncta), and autolysosomes (LC3- and LAMP1-positive puncta) (Figure 3C**-J**) at steady state and after 2 h of EBSS starvation. Consistent with our *in vivo* findings, the number of lysosomes was reduced in *spg11* KO, *zfyve26* KO and dKO MEFs already at nutrient conditions and further decreased after EBSS starvation (Figure 3K). Although the number of autophagosomes was increased in *spg11* KO, *zfyve26* KO and dKO MEFs at steady state, this difference was gone during EBSS starvation (Figure 3L). The number of autolysosomes was significantly increased in *spg11, zfyve26* KO and dKO MEFs under challenged conditions (Figure 3M) suggesting that the fusion of lysosomes with autophagosomes still occurs in the absence of ZFYV26, SPG11 or both. This assumption was corroborated by flux analysis with an mRFP-eGFP-LC3 reporter (ptfLC3), which labels autophagosomes in red and green and autolysosomes in red, because the GFP signal is quenched by the acidic pH in autolysosomes (**Fig. S3**).

Our findings are consistent with previous reports that the recycling of lysosomes from autolysosomes might be affected in SPG11 and SPG15 [13]. To visualize ALR we transiently expressed LAMP1-GFP in WT (**Movie S1**), *spg11* KO (**Movie S2**), *zfyve26* KO (**Movie S3**), and dKO (**Movie S4**) MEFs and quantified LAMP1-positive tubules longer than 2 µm between 8 and 9 h of starvation. In agreement with a similar defect in ALR, the ratio of cells with more than 5 tubules longer than 2 µm was decreased likewise in *spg11* KO and *zfyve26* KO MEFs (Figure 3N**-R**). Tubules longer than 5 µm were very rare events in both WT and in KO MEFs.

Altogether our *in vivo* and *in vitro* data support a similar defect of ALR upon disruption of either *Spg11* or *Zfyve26* or both.

### PI4K2A (phosphatidylinositol 4-kinase type 2 alpha) accumulates in autofluorescent deposits of *spg11* KO and *zfyve26* KO mice

**Figure 4.**
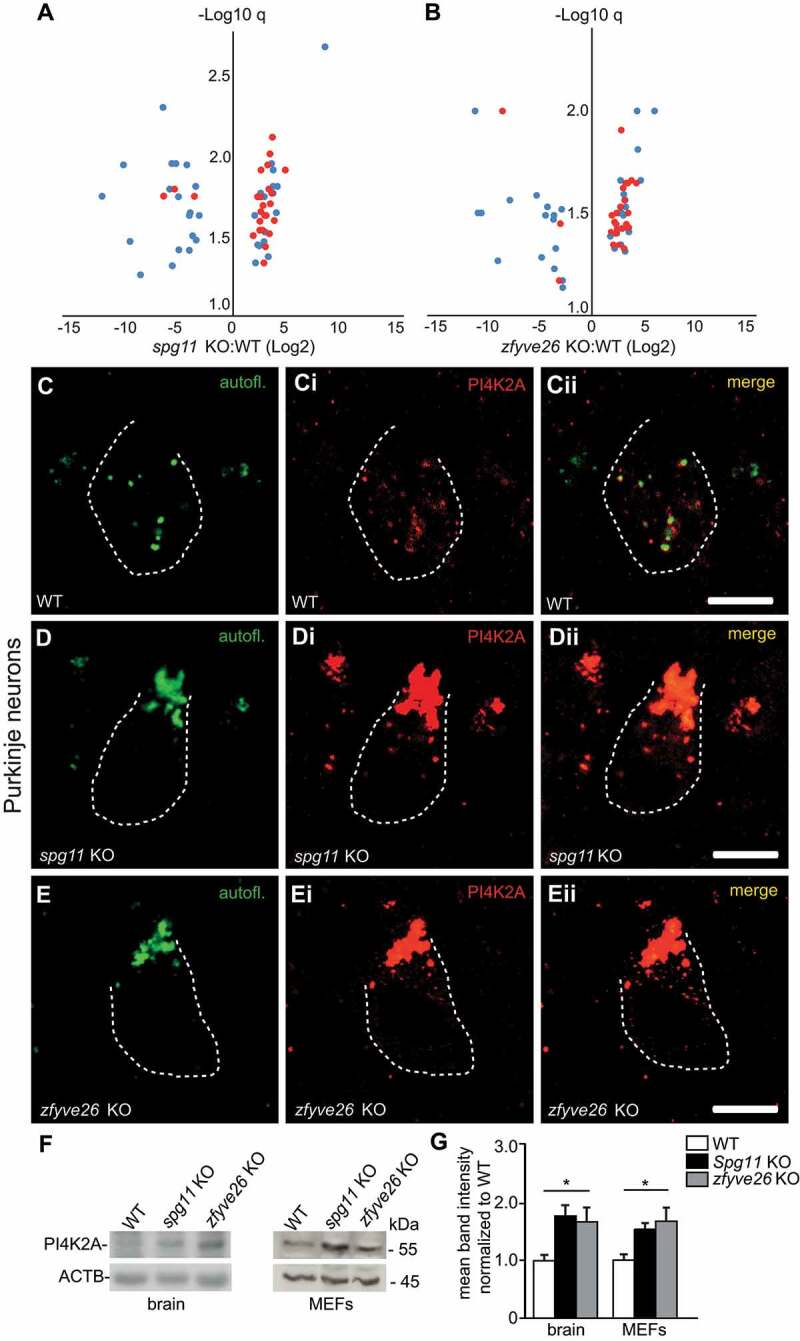
Increased abundance of PI4K2A in autofluorescent deposits of *spg11* KO and *zfyve26* KO mouse brains. (A and B) Autofluorescent material from brains of 6-months-old WT, *spg11* KO and *zfyve26* KO mice was isolated and analyzed by mass spectrometry. Volcano plot displaying log2 fold change of proteins vs. minimal limma q-value changed in the same direction in both s*pg11* KO and *zfyve26* KO samples compared to WT. Endomembrane proteins are shown in red. (**C-Eii**) Immunostainings of cerebellar sections confirm the accumulation of PI4K2A (red) in autofluorescent deposits (green) in Purkinje cells of 6-months-old *spg11* KO (D-Dii) and *zfyve26* KO (E-Eii) mice. Purkinje cell somata are marked by a dashed line. Scale bars: 5 µm. (F and G) Western blot analysis confirms the increased abundance of PI4K2A in brain lysates of 16-months-old *spg11* KO and *zfyve26* KO mice and in MEFs of respective genotypes. ACTB served as loading control (n = 5 experiments for MEFs and n = 4 mice per genotype for brain lysates; one-way ANOVA followed by Tukey’s Multiple Comparison Test; * p < 0.05). Error bars represent SEM

The drastic increase of autofluorescent material in *spg11* KO and *zfyve26* KO compared to WT neurons, prompted us to analyze the protein composition of these deposits. The material was isolated by density centrifugation from brains of 6-months-old WT, *spg11* KO and *zfyve26* KO mice as described previously [20]. Quantitative mass spectrometry identified more than 2,000 proteins in each sample (**Table S1**, raw data). There was a large overlap between proteins previously detected in rat and human lipofuscin [20] with proteins identified in deposits isolated from WT,*spg11* KO and *zfyve26* KO mice (**Table S2**). To get further insights into the pathophysiology of SPG11 and SPG15 we filtered for proteins that were significantly changed in the same direction in both *spg11* KO and *zfyve26* KO mice compared to WT (fold change log2 FC>1, q value -Log10 q > 1,3). This resulted in a list of 64 significantly down- or upregulated proteins (Figure 4A and **B; Table S3**). Notably, twenty-seven of these candidates were classified as endomembrane associated proteins with the cellular compartment tool of the Genome Ontology Consortium (GO) website [21]. The majority of these proteins were found to be enriched in KO samples (red dots, Figure 4A and **B; Table S3**, red lines). We also filtered for proteins that were only found in deposits from both *spg11* KO and *zfyve26* KO mice (**Table S4**). Out of the 15 proteins identified, PI4K2A was the most abundant in KO deposits.

To validate the accumulation of PI4K2A *in vivo* we stained brain sections from 6-months-old WT, *spg11* KO, and *zfyve26* KO mice. While there was only partial overlap with lipofuscin in aged WT mice (Figure 4C**-Cii**), PI4K2A colocalized with autofluorescent deposits in *spg11* KO (Figure 4D**-Dii**) and *zfyve26* KO (Figure 4E**-Eii**) Purkinje cells.

Immunoblot analysis showed a roughly 2-fold increase of PI4K2A abundance in the Triton X insoluble fraction of total brain lysates from 16-months-old *spg11* KO, *zfyve26* KO and dKO mice (Figure 4F and **G**). Significantly increased levels of PI4K2A were also observed in *spg11* KO and *zfyve26* KO MEFs (Figure 4F and **G**).

To further address why PI4K2A is increased upon disruption of *Spg11* or *Zfyve26*, we assessed *Pi4k2a* transcript abundance by quantitative PCR in MEFs, which was not increased in KO samples (**Fig. S4A**). We also transfected MEFs with a PI4K2A-GFP construct and quantified fluorescence intensity over time after inhibition of protein translation with Cycloheximide. While the decrease of fluorescence intensity in MEFs transfected with a GFP-construct alone did not differ between genotypes (**Fig. S4B**), the turnover of PI4K2A-GFP was impaired in *spg11* KO and *zfyve26* KO MEFs (**Fig. S4C**).

Taken together, we show that PI4K2A abundance is increased in brain lysates and in MEFs isolated from *spg11* KO or *zfyve26* KO mice, which may result from a delayed turnover of PI4K2A upon disruption of either *Spg11* or *Zfyve26*.

### PI4K2A abundance is increased in autolysosomes of pre-symptomatic s*pg11* KO and *zfyve26* KO mice

**Figure 5.**
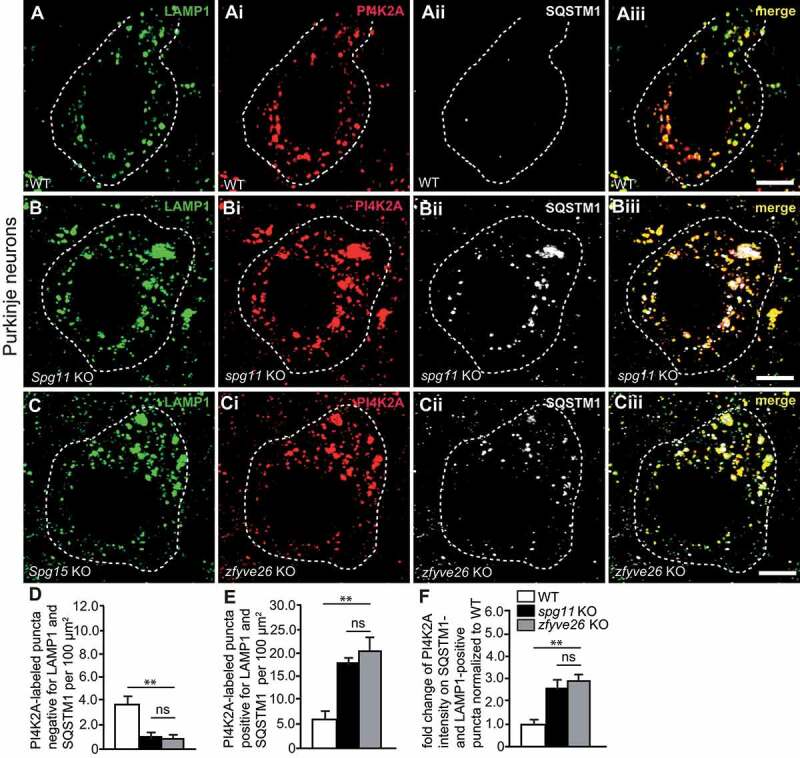
PI4K2A abundance is increased in autolysosomes of pre-symptomatic *spg11* or *zfyve26* KO mice. (**A-Ciii**) Stainings of brain sections of 2-months-old mice. Representative Purkinje cells in brain sections of WT (A-Aiii), s*pg11* KO (B-Biii) and *zfyve26* KO mice (C-Ciii) are shown stained for LAMP1 (green), PI4K2A (red) and SQSTM1 (white). Purkinje cell somata are marked by a dashed line. Scale bars: 5 µm. (D) PI4K2A-positive puncta negative for LAMP1 and SQSTM1 are decreased in *spg11* KO and *zfyve26* KO mice. (E) Puncta positive for PI4K2A, LAMP1 and SQSTM1 are increased in *spg11* KO and *zfyve26* KO mice. (F) Mean PI4K2A signal intensities are increased in SQSTM1- and LAMP1-positive puncta in Purkinje cells of *spg11* KO and *zfyve26* KO mice. More than 30 cells from n = 3 mice per genotype were analyzed (one-way ANOVA followed by Tukey’s Multiple Comparison Test; ** p < 0.01; ns: not significant). Error bars represent SEM

PI4K2A is a membrane-bound phosphatidylinositol 4-kinase, which mainly localizes to endosomes, the TGN [22], and lysosomes [23] and is recruited to autophagosomes during starvation [23]. To study whether the subcellular distribution of PI4K2A is altered upon disruption of *Spg11* or *Zfyve26*, we co-stained brain sections of 2-months-old pre-symptomatic KO mice for PI4K2A, LAMP1 and SQSTM1 (Figure 5A**-Ciii**). The number of PI4K2A-positive puncta negative for LAMP1 and SQSTM1 was decreased in Purkinje cells of *spg11* KO and *zfyve26* KO mice (Figure 5D). Nearly all autolysosomes defined as LAMP1- and SQSTM1-positive puncta also labeled for PI4K2A irrespective of the genotype and the number of PI4K2A-positive autolysosomes was elevated in KO mice (Figure 5E). Notably, mean signal intensities for PI4K2A on autolysosomes were more than doubled in KO mice (Figure 5F).

In summary, PI4K2A abundance is already increased in autolysosomes of pre-symptomatic *spg11* KO and *zfyve26* KO mice.

### PI4K2A modulates ALR

**Figure 6.**
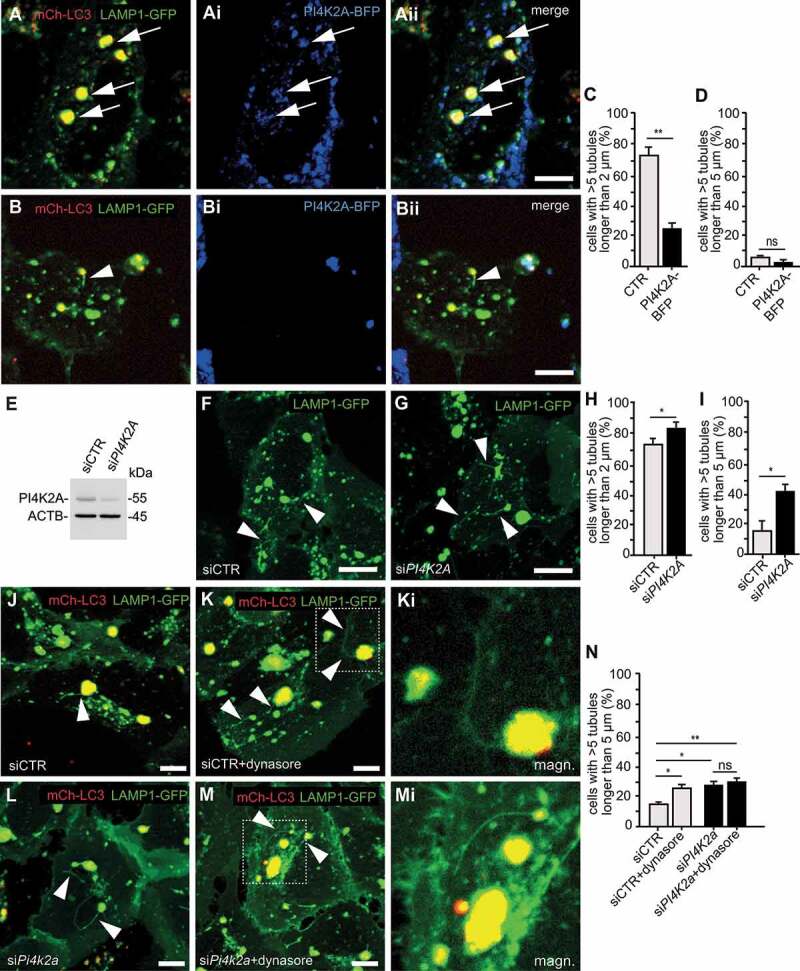
PI4K2A modulates autophagic lysosome reformation. (A-D) Overexpression of PI4K2A-BFP in U2-OS cells stably expressing LAMP1-GFP and mCherry-LC3 impairs ALR. Less tubulation of LAMP1-GFP (green) and mCherry-LC3 (red) labeled autolysosomes in a cell expressing PI4K2A-BFP (blue, marked with white arrows) (A-Aii) compared with a cell not expressing PI4K2A-BFP (B-Bii). Cells were starved with EBSS for 8–9 h. (C and D) Quantification of the ratio of cells with > 5 tubules longer than 2 µm or 5 µm in PI4K2A-BFP positive and negative (CTR) cells (at least 30 cells per group from n = 3 experiments; Student’s t-test: ** p < 0.01; ns: not significant). Scale bars: 10 µm. (E-I) SiRNA mediated knockdown of *PI4K2A* in U2-OS cells stably expressing LAMP1-GFP. (E) Immunoblot analysis of cells transfected with either scrambled siRNA (siCTR) or siRNA targeting *PI4K2A* (si*PI4K2A*) with ACTB as loading control. (F and G) Tubulation events (white arrowheads) in control (F) and knockdown (G) cells. Scale bars: 10 µm. (H and I) The ratio of cells with more than 5 LAMP1-positive tubules longer than 2 (H) or 5 µm (I) is increased upon knockdown of *PI4K2A* between 8 and 9 h of EBSS starvation (at least 30 cells per group from n = 3 experiments; Student’s t-test: * p < 0.05). (J-N) Inhibition of tubule scission with Dynasore resembles the tubulation defect observed upon knockdown of *PI4K2A* and does not aggravate the effect of *PI4K2A* knockdown on ALR (quantification of at least 30 cells per genotype from n = 3 independent experiments; one-way ANOVA followed by Newman-Keuls Multiple Comparison Test; *** p < 0.001; ns: not significant). Scale bars: 10 µm. Error bars represent SEM

We considered that the increased abundance of PI4K2A may be causally linked with the defect of ALR upon disruption of either *Spg11* or *Zfyve26*. To validate our hypothesis, we transfected U2-OS cells stably expressing LAMP1-GFP and mCherry-LC3 [24] with a construct encoding PI4K2A-BFP. Using live cell microscopy, we observed that PI4K2A-BFP was targeted to lysosomes and autolysosomes (Figure 6A**-Aii**). Recapitulating our findings in starved *spg11* or *zfyver26* KO MEFs, the ratio of cells with more than 5 tubules longer than 2 µm emerging from LAMP1 and LC3-labeled autolysosomes was drastically decreased in PI4K2A-BFP-positive cells between 8 and 9 h after initiation of EBSS starvation (Figure 6A**-C**). The ratio of cells with more than 5 tubules longer than 5 µm was low and did not change upon overexpression of PI4K2A-BFP (Figure 6D).

Next, we assessed whether the siRNA-mediated knockdown of PI4K2A in U2-OS cells affects ALR (Figure 6E**-I**). The knockdown of PI4K2A slightly increased the ratio of cells with more than 5 tubules longer than 2 µm (Figure 6H), while we observed a strong increase in the ratio of cells with more than 5 tubules longer than 5 µm emerging from autolysosomes (Figure 6I), suggesting that the scission of tubules might be affected. Along this line, inhibition of the GTPase DNM2, which is required for the scission of reformation tubules, with Dynasore also increased the ratio of cells with more than 5 tubules longer than 5 µm in starved U2-OS cells, while Dynasore did not increase this fraction any further upon knockdown of PI4K2A (Figure 6J**-N**).

**Figure 7.**
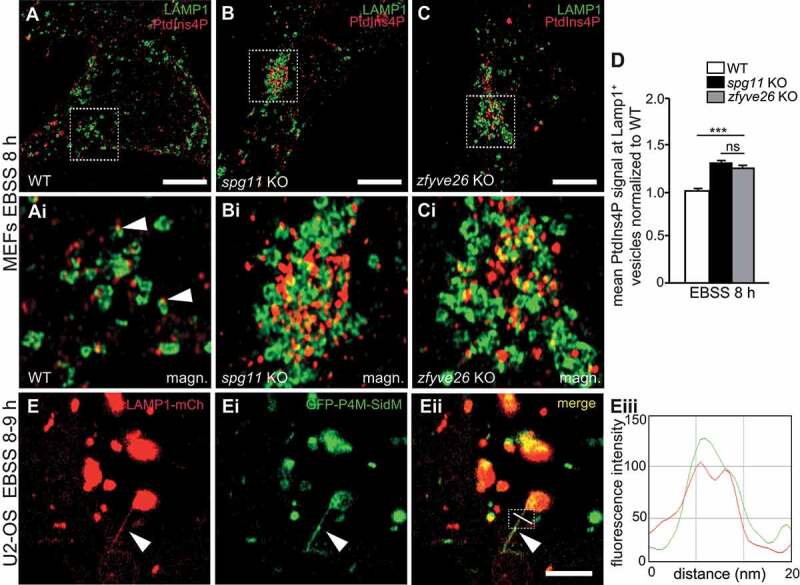
The abundance of PtdIns4P at LAMP1-positive structures is increased in *spg11* KO and *zfyve26* KO MEFs. (A-D) Compared to WT (A and Ai), PtdIns4P signals at LAMP1-positive puncta are increased in starved s*pg11* KO (B and Bi) and *zfyve26* KO (C and Ci) MEFs. More than 30 cells from n = 3 independent experiments were analyzed (one-way ANOVA followed by Tukey’s Multiple Comparison Test; *** p < 0.001; ns: not significant). Scale bar: 10 µm. Error bars represent SEM. (**E-Eiii**) LAMP1-positive reformation tubules of starved U2-OS cells stably expressing LAMP1-mCherry are labeled by a PtdIns4P-specific fluorescent sensor. Scale bar: 5 µm. (Eiii) Line scan as indicated in (Eii)

Because PI4K2A catalyzes the phosphorylation of phosphatidylinositol to PtdIns4P we immunostained WT and KO MEFs for PtdIns4P. Suggesting that higher levels of PI4K2A also entail increased levels of its product, PtdIns4P signals per cell were increased in *spg11* KO and *zfyve26* KO MEFs both at nutrient (**Fig. S5A-D**) and even more pronounced at starved conditions (**Fig. S5E-H**). We also quantified PtdIns4P signals at LAMP1-positive puncta after prolonged starvation. Notably, the PtdIns4P signals at LAMP1-positive puncta was increased in *spg11* KO and *zfyve26* KO MEFs (Figure 7A**-D**).

When we transfected U2-OS cells stably expressing LAMP1-mCherry with a PtdIns4P-specific fluorescent probe [25], the probe was clearly recruited to emerging LAMP1-positive tubules during ALR (Figure 7E**-Eiii, Movie S5**). Thus, our data support the assumption that PtdIns4P localizes to reformation tubules in accordance with a previous report [26]. Here, PtdIns4P may interact with a yet unidentified effector or may be further phosphorylated to phosphatidylinositol-4,5-bisphosphate (PtdIns [4,5]P_2_), which recruits clathrin and AP-2 (adaptor protein complex 2) [26] as well as KIF5B (kinesin family member 5B) [27] to budding and elongating reformation tubules.

**Figure 8.**
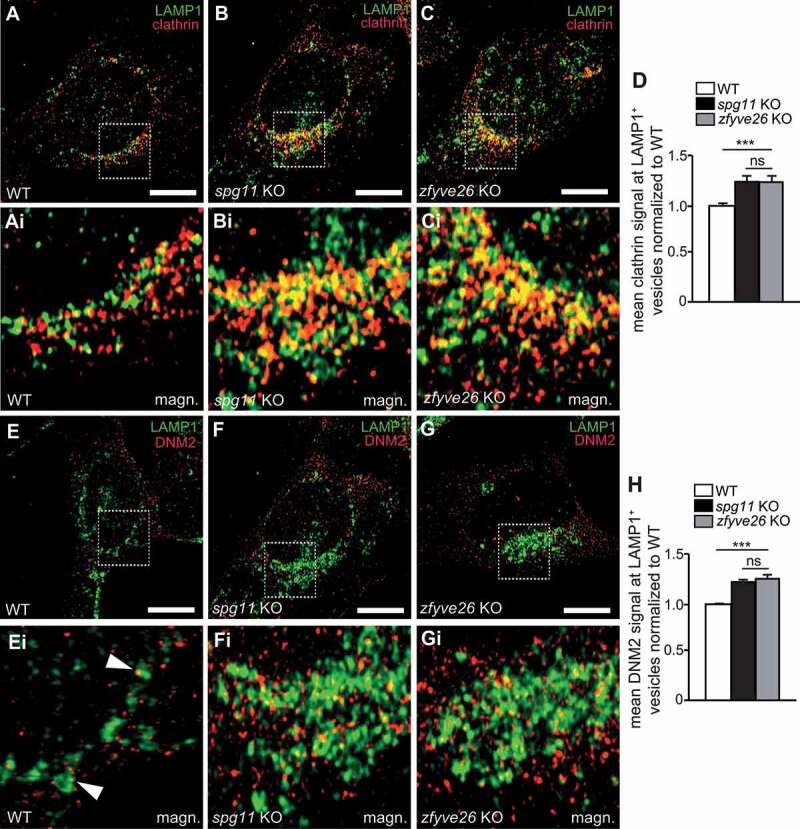
PI4K2A is involved in the recruitment of clathrin and DNM2 during ALR. (A-D) The association of clathrin with LAMP1-positive puncta is increased in starved *spg11* KO and *zfyve26* KO MEFs. Quantification of at least 30 cells per genotype from n = 3 independent experiments (one-way ANOVA followed by Newman-Keuls Multiple Comparison Test; *** p < 0.001; ns: not significant). Scale bars: 10 µm. (E-H) The association of DNM2 with LAMP1-positive puncta is increased in starved *spg11* KO and *zfyve26* KO MEFs. Quantification of at least 30 cells per genotype from n = 3 independent experiments (one-way ANOVA followed by Newman-Keuls Multiple Comparison Test; *** p < 0.001; ns: not significant). Scale bars: 10 µm. Error bars represent SEM

To obtain further mechanistic insights we also assessed the association of clathrin with LAMP1-positive puncta during ALR, which was increased in KO MEFs (Figure 8A**-D**). We also quantified the association of the GTPase DNM2, which can bind to PtdIns(4,5)P_2_ [28] and is required for the scission of proto-lysosomal vesicles from reformation tubules during the final step of ALR [29], with LAMP1-positive structures. Remarkably, the DNM2 signals associated with LAMP1-positive puncta were increased as well (Figure 8E**-H**). Overexpression of PI4K2A in WT MEFs enhanced the colocalization of clathrin (**Fig. S6A-C**) and DNM2 (**Fig. S6D-F**) with LAMP1-positive structures.

Taken together, our data suggest that the increased abundance of PI4K2A disturbs the tightly regulated balance between different PIP species and may thus disturb proper ALR.

## Discussion

Because SPG11 and SPG15 cannot be distinguished on clinical terms [3] and because the respective proteins were reported to partially colocalize [30], it has been speculated that SPG11 and SPG15 may be pathophysiologically related. The observation that SPG11 and ZFYVE26 co-precipitate in a complex with AP-5, was the first molecular correlate that both proteins act in the same pathway [4,5]. To further address this issue, we compared the phenotypes of *spg11* KO and *zfyve26* KO mice and crossed both lines to obtain dKO mice. Consistent with the assumption that SPG11 and ZFYVE26 act in the same molecular pathway, we did not observe any differences between the phenotypes of *spg11* KO and *zfyve26* KO mice. Moreover, the onset and the severity of symptoms were not aggravated upon simultaneous disruption of both. Notably, mice heterozygous for both KO alleles did not develop any gait abnormalities and did not show an accelerated accumulation of autofluorescent material in neurons as observed in KO mice. This is in contrast to data reported for zebrafish, in which the simultaneous partial knockdown of the *Spg11* and *Zfyve26* orthologues compromised motoneuron axon outgrowth, while the partial knockdown of either one alone was ineffective [31]. The conflicting data may either point to species differences, off-target effects or unspecific toxic effects of the Morpholino based knockdown, which is frequently observed [32]. Thus, our data argue against the assumption that HSP may be caused by the combination of a heterozygous mutation in one *SPG11* and one *ZFYVE26* allele [31]. To our knowledge such patients have not been reported to date.

Because of its predicted structure it is assumed that SPG11 may be a component of the outer part of the AP-5-containing coat, possibly acting as a membrane-deforming scaffold, while ZFYVE26 may target the complex to membranes enriched in the endosomal phosphoinositide PtdIns3P via its FYVE-domain [9]. M6PR (mannose-6-phosphate receptor, cation dependent) and SORT1/sortilin 1 both interact with ZFYVE26 and may thus represent cargo receptors of AP-5 [6]. Impaired retrieval of proteins from late endosomes to the Golgi possibly involving SORT1 may thus explain the redistribution of Golgi-related proteins to late endosomes/lysosomes upon disruption of AP-5 [6,18].

Apart from its interaction with AP-5, ZFYVE26 was also shown to interact with BECN1/Beclin 1 [11], a central regulator of autophagosome maturation. Notably, this interaction was disturbed in disease causing ZFYVE26 variants [33] and caused an accumulation of immature autophagosomes upon starvation in patient derived fibroblasts [11,34]. Both SPG11 and ZFYVE26 interact with RAB5A (RAB5A, member RAS oncogene family) and RAB11 [35], two proteins involved in endosome maturation and trafficking. Only disease associated ZFYVE26, but not SPG11 variants affected RAB protein interactions and impaired the fusion of endosomes and autophagosomes, thus causing a more severe autophagy defect compared to *SPG11* [35]. This is in contrast to our data, which do not support a fusion defect of autophagosomes with lysosomes in *zfyve26* KO MEFs. Possibly, the disease-associated variants studied may not represent full KOs and differences may thus reflect variant-specific effects. Although autophagosome numbers were elevated in *spg11* KO, *zfyve26* KO and dKO MEFs at base line, the numbers did not differ from WT at starved conditions. Instead, we found an accumulation of autolysosomes in starved KO MEFs, which resembles previous reports for the knockdown of *SPG11* or *ZFYVE26* in HeLa cells [12]. A fusion of autophagosomes and lysosomes in *spg11* KO and *zfyve26* KO MEFs was also evident in our flux analysis with a LC3 tandem reporter. As suggested by our LAMP1 and LC3 staining of brain sections, a depletion of lysosomes and an accumulation of autolysosomes also apply *in vivo* upon disruption of *Spg11* or *Zfyve26.*

To get further clues about the pathophysiology of SPG11 and SPG15, we performed an unbiased mass spectrometry analysis of autofluorescent material isolated from WT, *spg11* KO and *zfyve26* KO brains. We identified high levels of PI4K2A in material isolated from both KO mice, while PI4K2A was not detected in autofluorescent material isolated from control mice. Immunoblot analysis of brain lysates and MEFs suggested a significant increase of PI4K2A abundance upon disruption of *Spg11* or *Zfyve26*. PI4K2A is a membrane-bound phosphatidylinositol 4-kinase, which mainly localizes to LAMP1-positive endosomes and lysosomes [36] and the TGN [22,37] and is recruited to autophagosomes during starvation [23,38]. Here, we show that PI4K2A is also present in autolysosomes and our analyses suggest that the abundance of PI4K2A in autolysosomes is increased upon disruption of either *Spg11* or *Zfyve26*. As suggested by our immunostainings in MEFs this likely changes local levels of its product PtdIns4P. PtdIns4P plays important roles in exocytosis, Golgi function, protein sorting and membrane trafficking [39,40] and may also affect ALR as the terminal step of autophagy.

To mimic our observation that PI4K2A abundance is increased upon disruption of either *Spg11* or *Zfyve26* we overexpressed PI4K2A in control U2-OS cells. Of note, this caused a reduction of LAMP1-positive tubules upon prolonged starvation. Overexpression of PI4K3B (phosphatidylinositol 4-kinase 3 beta), another lipid kinase contributing to the production of PtdIns4P, also decreased the number of lysosomal tubules, while tubules were increased upon PI4K3B knockdown [41]. Likely, the role of these kinases for ALR is complex, because PtdIns4P can be phosphorylated to PtdIns(4,5)P_2_, which recruits clathrin to lysosomal budding sites in an AP-2 dependent process [26] and can also bind DNM2, which is required for tubule scission [29]. Indeed, we found that the association of PtdIns4P and clathrin and DNM2 with LAMP1-postivie structures was increased in *spg11* KO and *zfyve26* KO MEFs. Notably, we observed the same effect upon overexpression of PI4K2A in WT MEFs.

Of note, a gene trap-based mouse model, which lacks the catalytic domain of PI4K2A, also accumulated autofluorescent deposits in neurons, which subsequently degenerated [42]. It thus appears that local PtdIns4P concentrations on autolysosomes have to be tightly controlled in both directions to allow autophagy and ALR. This is further highlighted by recent findings for the lipid phosphatase INPP5K (inositol polyphosphate-5-phosphatase K), which hydrolyzes PtdIns(4,5)P_2_ to PtdIns4P. Its disruption caused the accumulation of PtdIns(4,5)P_2_ on autolysosomes and prevented the disengagement of clathrin from reformation tubules [43], thus suppressing ALR. A similar mechanism may also apply to DNM2 and tubule scission further contributing to defective ALR. Thus, the bidirectional interconversion of PtdIns4P/PtdIns(4,5)P_2_ on autolysosomes appears to be a critical event for lysosomal homeostasis.

So far, we do not know why PI4K2A accumulates in LAMP1-positive compartments in the absence of SPG11 or ZFYVE26. This cannot be explained at the transcriptional level, because *Pi4k2a* transcript abundance was not changed upon disruption of either *Spg11* or *Zfyve26*. Analysis of PI4K2A protein abundance after inhibition of translation rather suggests that the turnover of PI4K2A is delayed. Notably, PI4K2A is a target of the E3 ubiquitin ligase Itch, which localizes to the TGN and endosomal compartments [44]. Since SPG11 and ZFYVE26 interact with AP-5, it is tempting to speculate that the retrieval of PI4K2A from autolysosomes is impaired upon disruption of AP-5. Notably, mutations in AP5Z1 are associated with SPG48 [4]. *ap5z1* KO mice also accumulate autolysosome-related autofluorescent material in neurons and show a defect of autophagic flux [18].

## Material and methods

### Animals

All animal experiments were approved by the “Thüringer Landesamt für Lebensmittelsicherheit und Verbraucherschutz” (TLLV) in Germany (Approval number: 02–039-14). *spg11 zfyve26* double knockout dKO mice were obtained by mating *spg11* KO [14] and *zfyve26* KO [7] mice. Studies were performed with mice on a C57BL/6 background. Mice were housed with a 12 h light/dark cycle and fed on a regular diet *ad libitum.*

### Behavioral analysis

For gait analysis mice were trained to walk on a horizontal 20-cm elevated plastic beam (1,000-mm long, either 38-mm broad for cohorts between 8 and 12 months of age or 48-mm broad for mice with an age between 2 and 12 months of age) leading to their home cage as described [7]. After the initial learning phase, the foot-base-angle at toe-off positions of the hind-paws was measured with ImageJ using single video frames from recordings of beam walking mice.

### Histological analysis

Immunohistochemistry was done on formalin fixed free floating sections as described previously [45]. Cortical motoneuron and Purkinje cell numbers were quantified on 40-µm sagittal brain sections after staining of brain sections with an antibody directed against RBFOX3 and CALB1. For statistical analysis the number of RBFOX3-positive neurons per 10,000 µm^2^ area per layer from motocortex and/or Purkinje cells per 1,000 µm distance along the Purkinje cell layer was counted from 3 different brain sections from 3 mice per genotype. Images of the sagittal sections of wild-type and knockout brains were taken with a confocal scanning fluorescence microscope (Zeiss LSM 880) with Airyscan using a Plan-Apochromat 63x/1.4 oil DIC M27 objective. Neurons and intracellular vesicles were quantified manually with the cell counter plugin and the area measurement tool of the ImageJ online software. For spectral analysis the fluorescent signals of deposits and the Cy5 secondary antibodies were recorded and further analyzed by linear unmixing as described previously [7].

### Western blotting

For immunoblot analysis, brain samples were homogenized in a buffer containing 25 mM sucrose (Sigma-Aldrich, S7903), 50 mM Tris-HCl, pH 7.4, 1 mM EDTA, completed with protease inhibitor mix (Roche, 04693124001). The homogenates were centrifuged at 500 g at 4°C for 10 min. The supernatants were collected and mixed with a buffer containing 25 mM sucrose, 50 mM Tris-HCl, pH 7.4, 1 mM EDTA, 1% Triton X-100 (Sigma-Aldrich, T9284) completed with proteinase inhibitors and gently rotated for 1 h at 4°C (i.e., Triton X-100 soluble fraction). After centrifugation at 13,000 g and 4°C for 15 min the pellets were dissolved in 1% (w:v) SDS-PBS (137 mM NaCl, 2.7 mM KCl, 10 mM Na_2_HPO_4_, 1.8 mM KH_2_PO_4_) buffer (i.e., Triton X-100 insoluble fraction). The samples were denatured at 95°C in Laemmli buffer and separated in 4–20% pre-cast gradient gels (Bio-Rad). After transfer on a PVDF membrane (GE Healthcare Amersham, GE10600023), 2.5% (w:v) milk powder (Santa Cruz Biotechnology, sc-2325) with 2.5% (w:v) BSA (Serva, 11930.03) in TBS-T buffer (Tris-buffered saline (137 mM NaCl, 25 mM Tris-Base, 2.7 mM KCl, pH 7.4) with Tween 20 (Sigma-Aldrich, P9416) was used in order to block the membrane. Primary and secondary antibodies were incubated in blocking solution. For detection of proteins the ECL Western Blotting Detection System (Bio Rad, 170–5061) was used. For autophagy studies mouse embryonic fibroblasts (MEFs obtained from *spg11* KO, *zfyve26* KO and dKO mice, as described above) were either incubated for 6 h with 1 µM Torin 1 (Merck Millipore, 475991) to initiate or 100 nM bafilomycin A_1_ (Merck Millipore, 19–148) to block autophagy and subsequently lysed in a buffer containing 50 mM Tris-HCl pH 8.0, 120 mM NaCl, 0.5% NP-40 (VWR, A1694.0250), 2 mM EDTA completed with protease inhibitor mix (Roche, 4693159001). The lysates were rotated for 30 min and centrifuged at 10,000 g at 4°C. The resulting supernatant was denatured in sample buffer for 5 min at 95°C and separated in 8–15% polyacrylamide pre-cast gradient gels. 3% BSA in TBS-T (MEFs) or 10% skimmed milk served as blocking solution.

### Mass spectrometric analysis of deposits

The deposits for mass spectrometry were isolated from total brain lysates of 6-months-old mice (n = 6 per genotype) as described [20] and used for the quantitative proteomics modified from [46,47]. In brief, protein solutions were reduced with 20 mM DTT at 55°C for 20 min and loaded onto centrifugal filter units with a 10 kDa cutoff modified Polyethersulfone (PES) membrane (Pall, OD010C34). The buffer was exchanged by centrifugation and addition of 20 mM triethylammonium bicarbonate (TEAB) (Sigma-Aldrich, T7408), 0.5% sodium deoxycholate (SDC) (Sigma-Aldrich, 30968). Alkylation of thiol groups was done with 40 mM acrylamide (Sigma-Aldrich, A9099) for 20 min at room temperature. After another buffer exchange 1 µg trypsin (Promega, V5111) was added in 20 mM TEAB, 0.5% SDC in a total volume of 50 µl. Digestion proceeded overnight at 37°C. Peptides were collected and SDC was precipitated with TFA (VWR, 85049.001P) (2.5% final). Remaining SDC was removed by phase transfer with equal volume of ethyl acetate. Peptides were vacuum concentrated and labeled with amine-reactive, 6-plex tandem mass tag reagents (Thermo Fisher Scientific, 90061) according to manufacturer’s instructions. The labeling reaction was quenched by addition of 5% hydroxylamine (Sigma-Aldrich, 467804). Labeled peptides were pooled and desalted on Oasis HLB cartridges (Waters GmbH, WAT094225). Eluates containing 70% acetonitrile (AppliChem, 7218811612), 0.1% formic acid (FA) (VWR, 85048.001P) were dried and fractionated into 12 fractions by isoelectric point with an Offgel fractionator (Agilent Technologies). Peptide fractions were dried and stored at −20°C.

Peptides were dissolved in 10 µl 0.1% FA (solvent A). 1.5 µl were injected onto a C18 trap column (20 mm length, 100 µm inner diameter, ReproSil-Pur 120 C18-AQ, 5 µm, Dr. Maisch GmbH) made in-house. Bound peptides were eluted onto a C18 analytical column (200 mm length, 75 µm inner diameter, ReproSil-Pur 120 C18-AQ, 1.9 µm). Peptides were separated during a linear gradient from 2% to 35% solvent B (90% acetonitrile, 0.1% formic acid) within 120 min at 300 nl/min. The nanoHPLC was coupled online to an LTQ Orbitrap Velos mass spectrometer (Thermo Fisher Scientific). Peptide ions between 330 and 1600 m/z were scanned in the Orbitrap detector with a resolution of 30,000 (maximum fill time 400 ms, AGC target 10^6^). The 20 most intense precursor ions (threshold intensity 5,000, isolation width 1.1 Da) were subjected to higher collision dissociation (HCD, stepped collision energy 32, 42, 52%) and analyzed in the Orbitrap detector (R = 7500). Fragmented peptide ions were excluded from repeat analysis for 17 s. Raw data processing and analysis of database searches were performed with Proteome Discoverer software 2.1.1.21 (Thermo Fisher Scientific). Peptide identification was done with an in house Mascot server version 2.5 (Matrix Science Ltd). MS2 data were searched against the Uniprot mouse reference proteome (release 2016_11). Precursor ion m/z tolerance was 9 ppm, fragment ion 20 mmu. a-, b- and y-ion series were included. Tryptic peptides with up to two missed cleavages were searched. Propionamide modification of cysteines and TMT-modification of peptide N-termini were set as static modifications. Oxidation of methionine, protein N-terminal acetylation, and TMT-modification of lysines were allowed as dynamic modifications. Mascot results were assigned q-values by the percolator algorithm [47] version 2.05 as implemented in Proteome Discoverer. Spectra of peptide spectrum matches (PSMs) with q > 0.01 were sent to a second round of database search with semitryptic enzyme specificity (one missed cleavage allowed). Dynamic modifications were the same as above plus propionamide (cysteine), di-Gly on lysine, and TMT on N-termini. Proteins were included if at least two peptides were identified with q ≤ 0.01. False positive rates were estimated to be 1.0%, 1.2%, and 4.5% on PSM, peptide, and protein level respectively. In order to reduce the reporter ion compression bias PSMs with >30% co-isolation were excluded from quantification. Only unique peptides were included in protein quantification. 2,088 proteins with intensities in ≥ 2 biological replicates were used for median normalization. KO:WT ratios of proteins with ≥ 3 replicates were sent to limma and rank product tests with correction for multiple testing [48]. A threshold of corrected p-value ≤ 0.05 defined significant regulation.

### Antibodies and DNA constructs

The following commercially obtained antibodies were used: rat anti-LAMP1, 1:250 for immunofluorescence (IF) and 1:500 for western blotting (WB) (BD Pharmigen, 553792); mouse anti-RBFOX3/NeuN, 1:500 (Millipore, MAB377); mouse anti-SQSTM1/p62, 1:500 for IF and 1:1,000 for WB (Abcam, ab56416); mouse anti-PI4K2A, 1:50 for IF and 1:500 for WB (Santa Cruz Biotechnology, sc-390026); and rabbit anti-PtdIns4P, 1:200 (Echelon biosc., Z-P004) mouse anti-ACTB/β-actin (Abcam, ab6276), 1:5,000 and mouse ant-GFP, 1:2,000 (Millipore, MAB3580); rabbit anti-LC3B, 1:1,000 for WB and 1:200 for ICC (Cell Signaling Technology, 2775S); mouse anti LC3B, 1:50 for IHC (Nanotools, 0231–100/LC3-5 F10); rabbit anti-GAPDH, 1:1,000 for WB (Santa Cruz Biotechnology, sc-25778); mouse anti-clathrin (Abcam, ab2731) 1:1,000 for IF; rabbit anti-DNM2/Dynamin-2 1:15,000 (R2641, a generous gift of IGBMC). Horseradish peroxidase-labeled secondary antibodies for western blotting: goat anti-rabbit (NA9340) and goat anti-mouse (NA9310), both 1:2,000 (Amersham Bioscience) and goat anti-rat, 1:1,000 (Santa Cruz Biotechnology, sc-2032).

The following fluorescently labeled secondary antibodies were used from Thermo Fisher Scientific: goat anti-mouse-Alexa Fluor 488 (A11029) and 546 (A11030), goat anti-rabbit Alexa Fluor 488 (A11008) and 546 (A11035), 1:1,000; goat anti-rabbit (A10523), goat anti-mouse (A10524) and goat anti-rat Cy5 (A10525), 1:1,000; goat anti-mouse IgM chain specific Cy5 conjugated (Merk, AP500S), goat anti-mouse IgG1 Alexa Fluor 546 (Thermo Fisher Scientific, A-21123) and goat anti-mouse IgG2a, Alexa Fluor 647 (Thermo Fisher Scientific, A-21241). Nuclei were stained with Hoechst-33258, 1:10,000 (H3569).

The LAMP1-GFP plasmid was a gift from Esteban Dell’Angelica (Addgene, 34831) [49]. PI4K2A-GFP, and EGFP-x2P4M-SidM plasmids were provided by Dr. Tamas Balla (National Institutes of Health, Bethesda, USA) [25]. For cloning of the PI4K2A-BFP DNA construct, the C-terminal GFP of PI4K2A-GFP was exchanged by BFP sequence using the EcoRI and NotI restriction sites. RFP-LC3 was a kind gift of Dr. Christoph Kaether (Fritz-Lipmann-Institute Jena, Germany). The ptfLC3 plasmid [50] was a gift from Tamotsu Yoshimori (Addgene, 21074).

### Knockdown studies

siRNAs directed against human *PI4K2A* transcripts was purchased from Santa Cruz Biotechnology (sc-90773) and used according to the manufacturer. The knockdown was verified by immunoblotting.

### Cell culture and Time-lapse imaging

U2-OS LAMP1-GFP or LAMP1-mCherry single *knockin* and U2-OS mCherry-LC3/LAMP1-GFP double *knockin* cell lines were provided by Dr. Ian G. Ganley from the University of Dundee, which were previously characterized in [24]. Mouse embryonic fibroblasts (MEFs) were prepared from E13.5 mouse pups as described [45]. The cells were cultured in the following growth medium: Dulbecco’s Modified Eagle medium (DMEM, Gibco, 31966–021) supplemented with 10% fetal bovine serum (biowest, S1810-500) and 1% penicillin streptomycin (Gibco, 15070–063) and maintained in a humidified atmosphere with 5% CO_2_ and 37°C. For autophagic flux analysis by western blotting MEFs were cultured onto 6-well dishes at a density of 300,000 per well in growth medium overnight. At day 2 cells were washed 3 times with ice-cold PBS and then treated with EBSS, EBSS supplemented with 1 µmol Torin (Calbbiochem, 475991) and/or additionally with 100 nM bafilomycin A_1_ (Sigma-Aldrich, 19–148) for 6 h. After treatment, cells were washed twice on ice in ice-cold PBS and lysed directly by addition of RIPA lysis buffer. Samples were sonicated, boiled and subjected to SDS-PAGE and western blotting as described previously [7].

For detection of lysosomal tubules, MEFs and/or U2-OS cells were plated on 42 mm coverslips (PeCon, 000380) in 6 cm plates and transfected after 24 h with 3 µg of each plasmid DNA: LAMP1-GFP, PI4K2A- BFP, or PI4K2A siRNA using Lipofectamine 3000 (Thermo Fischer, L3000008) for 48 h. To induce autophagy, cells were incubated with EBSS medium (Thermo Fischer, 24010043) for 8–9 h. For inhibition of DNM2, 40 µM Dynasore solution (Millipore, 324410) was added 2 h before the acquisition. Tubulation events were visualized with a fluorescence microscope (CellObserver Z1, Zeiss) equipped with an incubation chamber (37°C, 5% CO_2_). Time-lapse images were acquired every 2 s for 3 min using the ApoTome mode and the 40x/1.2 W objective. Number and length of tubules were evaluated for at least 30 cells of each genotype in 3 independent experiments using ImageJ (National institutes of health).

To detect PtdIns4P in live cell microscopy experiments, U2-OS LAMP1-mCherry cells were plated in a µ-Slide 8 well chamber slide (IBIDI, 80,826) and after 24 h transfected with 0.5 µg pEGFP-x2P4M-SidM [25]. After 8 h treatment with EBSS, tubulation events were visualized with a confocal scanning fluorescence microscope (Zeiss LSM 880) with Airyscan equipped with an incubation chamber (37°C, 5% CO_2_, Tokai hit WSBX incubator) using a Plan-Apochromat 63x/1.4 oil DIC M27 objective.

### Immunocytochemistry

Cells were seeded on 14 mm coverslips and fixed in 4% PFA or 100% ice-cold methanol (LC3 staining) for 10 min. Cells were washed three times, permeabilized with 0.25% Triton X-100 for 10 min. and blocked using 5% normal goat serum (BIOZOL, S-1000) for 1 h at room temperature. Coverslips were incubated overnight with primary antibodies diluted in blocking solution at 4°C. The next day the coverslips were washed three times and incubated with secondary antibodies conjugated with a fluorophore for 1 h at room temperature. After washing three times with 1xPBS cells were stained with Hoechst (1:10,000, Invitrogen, H3569) and mounted with fluoromount mounting medium (BIOZOL, SBA-0100-01) Stainings with for PtdIns4P were perfomed as described previously [51]. Images were acquired with a confocal scanning fluorescence microscope (Zeiss LSM 880) with Airyscan using the Plan-Apochromat 63x/1.4 oil DIC M27 objective, and further analyzed in ImageJ. For the particle colocalization analyses the ComDet plugin of ImageJ was used.

### Flow Cytometry

To investigate the degradation of PI4K2A in MEFs cells were transfected with PI4K2A-GFP or empty vector (pEGFP-N1). After 48 h cells were harvested or treated with 10 µg/ml Cycloheximide (Sigma-Aldrich, C4859) for 4 h. Cells were fixed in 4% PFA and stored at 4°C until measurement. Flow cytometry was performed and analyzed with the BD Accuri C6 Plus Flow cytometer and software (BD biosciences) according to manufacturer’s protocol. Prior the analysis of GFP fluorescence, cell debris and aggregates were excluded with pre-gating on FSC/SSC.

### Quantitative reverse transcription PCR

RNA was isolated from MEFs using preGOLD TriFast (VWR, 30–2010) according to the manufacturer’s protocol. Two-step quantitative reverse transcription PCR (qRT-PCR) was performed with the following specific primers: *Pi4k2a*-F: CTCCCTGAGAACACGAACCG *Pi4k2a*-R: ATCACCCAATCTGTGTCCCG; *Actb*-F: AGAGGGAAATCGTGCGTGAC *Actb*-R: CAATAGTGATGACCTGGCCGT.

### Statistical analysis

The results were blinded to experimenter and were analyzed in Graphpad Prism 6 (Graphpad Software Inc). Data in graphs are presented as mean ± SEM. In some of the results, values are normalized on the mean of control samples, which is represented as 1. The differences between many groups were determined by using of one-way ANOVAs followed by Tukey’s Multiple Comparison test or by Newman-Keuls Multiple Comparison test. The differences between many groups and repeated measurements were estimated by two-way ANOVAs with Bonferroni posthoc test. For differences between 2 groups statistical significance was determined by using of unpaired two-tailed Student’s t-tests. The significance values are represented as; p* < 0.05, p** < 0.01 and p*** < 0.001. Non-significant p values are represented as: ns.

## Supplementary Material

Supplemental Material
