# Supplementary material for: Mouse models for hereditary spastic paraplegia uncover a role of PI4K2A in autophagic lysosome reformation
Source: Autophagy. 2021 Mar 9;17(11):3690–706. doi: 10.1080/15548627.2021.1891848 (PMC8632344; doi:10.1080/15548627.2021.1891848)
Supplement: Supplemental Material [file KAUP_A_1891848_SM4373.zip › Khundadze et al figure legends supplemental material.docx]

**Supplementary material**

**Figure S1**. Autofluorescence and SQSTM1 abundance is likewise increased in Purkinje cells of 16-months-old *spg11* KO, *zfyve26* KO and *spg11 zfyve26* double-KO mice (dKO). (**A-C**) Compared to WT (A) Purkinje cell autofluorescence (green, excitation at 488 nm) is not increased in 2-months-old mice heterozygous for the *Spg11* and *Zfyve26* (dHET) allele (B) (more than 30 cells per genotype; n=3 mice per genotype; one-way ANOVA followed by Tukey's Multiple Comparison Test; ns: not significant). Scale bars: 5 μm. (**D-Gii**) Autofluorescent material in Purkinje cells of 16-months-old WT mice (D-Dii) rarely co-localizes with SQSTM1, while the large autofluorescent deposits in *spg11* KO (E-Eii), *zfyve26* KO (F-Fii) and double (G-Gii) KO mice regularly stain for SQSTM1. Purkinje cell somata are marked by a dashed line. Scale bars: 5 µm. (**H** and **I**) Quantification of autofluorescence and of SQSTM1 signal intensities. More than 30 cells from 3 mice per genotype were analyzed (one-way ANOVA followed by Tukey's Multiple Comparison Test; *** p<0.001; ns: not significant). Error bars represent SEM.

**Figure S2.** Less lysosomes in Purkinje cells of *spg11* KO, *zfyve26* KO and *spg11 zfyve26* double KO mice. (**A-F**) Lysosome numbers are likewise reduced in Purkinje cells of 2-months-old *spg11* KO, *zfyve26* KO and double KO mice. Representative Purkinje cells in brain sections of WT (A-Aii), *spg11* KO (B-Bii), *zfyve26* KO (C-Cii) and double KO (D-Dii) mice stained for LAMP1 (green) and SQSTM1 (red). Purkinje cell somata are marked by a dashed line. (E and F) Quantification of lysosomes (E) defined as LAMP1-positive and-negative puncta and autolysosomes (F) defined as LAMP1-positive and SQSTM1-positive puncta. More than 30 cells from 3 mice per genotype were analyzed (one-way ANOVA followed by Tukey's Multiple Comparison Test; * p<0.05; ns: not significant). Scale bars: 5 µm. Error bars represent SEM.

**Figure S3.** The fusion of lysosomes with autophagosomes occurs in *spg11* KO, *zfyve26* KO and *spg11 zfyve26* double KO (dKO) MEFs. (**A-I**) Cells were transfected with the mRFP-eGFP-LC3 reporter and the ratio between total mRFP and eGFP fluorescence calculated per cell at steady-state (A-D) and 6 h after induction of EBSS starvation (E-H). Scale bars: 10 µm. (I) Because eGFP signals are quenched in acidic compartments, an increased mRFP:eGFP ratio upon starvation indicates fusion of autophagosomes with lysosomes (quantification of at least 30 cells per group from n=3 experiments; one-way ANOVA; ** p<0.01). Error bars represent SEM.

**Figure S4.** Delayed turnover of PI4K2A-GFP upon disruption of either *Spg11* or *Zfyve26*. (**A**) Quantitative real-time PCR suggests that *Pi4k2a* transcript abundance normalized to *Actb* is not changed in *spg11* KO and *zfyve26* KO MEFs (quantification from 3 replicates each and n=3 independent experiments; one-way ANOVA; ns: not significant). (**B**) FACS analysis of mean GFP fluorescence intensity in MEFs transfected with a GFP construct before and 4 h after addition of Cycloheximide normalized to baseline (n=3 independent experiments; one-way ANOVA; *** p<0.001). (**C**) FACS analysis of mean GFP fluorescence intensity in MEFs transfected with a PI4K2A-GFP construct before and 4 h after addition of Cycloheximide (n=3 independent experiments; one-way ANOVA; *** p<0.001; ns: not significant). Error bars represent SEM.

**Figure S5.** PtdIns4P signals are increased in *spg11* KO and *zfyve26* KO mouse embryonic fibroblasts. (**A-D**) Compared to WT (A) PtdIns4P signals are significantly increased in *spg11* KO (B) and *zfyve26* KO (C) MEFs at steady state (more than 30 cells per genotype; n=3 experiments; one-way ANOVA followed by Tukey's Multiple Comparison Test; * p<0.05). (**E-H**) Upon induction of autophagy by 4 h of EBSS starvation, PtdIns4P signals decrease in WT (E) but remain elevated in *spg11* KO (F) and *zfyve26* KO (G) MEFs (more than 30 cells per genotype; n=3 experiments; one-way ANOVA followed by Tukey's Multiple Comparison Test; *** p<0.001). Scale bars: 5 µm. Error bars represent SEM.

**Figure S6.** PI4K2A-BFP overexpression in WT MEFs increases the recruitment of clathrin and DNM2 to LAMP1-positive structures. (**A-C**) Co-stainings for LAMP1 and clathrin in control MEFs (A-Aii) and after overexpression of PI4K2A (B-Bii). (C) Quantification of at least 30 cells per genotype from n=3 independent experiments (Student’s t-test; *** p<0.001). (**D-F**) Co-stainings for LAMP1 and DNM2 in control MEFs (D-Dii) and after overexpression of PI4K2A (E-Eii). (F) Quantification of at least 30 cells per genotype from n=3 independent experiments (Student’s t-test; *** p<0.001). Scale bars: 10 µm. Error bars represent SEM.

**Table S1.** Full list of proteins identified in autofluorescent material isolated from 6-months-old WT, *spg11* KO and *zfyve26* KO mice.

**Table S2.** List of proteins from *spg11* KO and *zfyve26* KO deposits, which have also been reported for human and rat lipofuscin.

**Table S3.** List of significantly regulated proteins identified in autofluorescent deposits isolated from *spg11* KO and *zfyve26* KO brains.

**Table S4.** List of proteins identified only in autofluorescent deposits isolated from 6-months-old *spg11* KO and *zfyve26* KO brains.

**Movie S1.** LAMP1-GFP-positive tubules in WT MEF

**Movie S2.** LAMP1-GFP-positive tubules in *spg11* KO MEF

**Movie S3.** LAMP1**-**GFP-positive tubules in *zfyve26* KO MEF

**Movie S4.** LAMP1**-**GFP-positive tubules in dKO MEF

**Movie S5.** LAMP1**-**mCherry- and PtdIns4P-positive tubules in U2-OS cells
