# Supplementary figures and images for: Mouse models for hereditary spastic paraplegia uncover a role of PI4K2A in autophagic lysosome reformation
Source: Autophagy. 2021 Mar 9;17(11):3690–706. doi: 10.1080/15548627.2021.1891848 (PMC8632344; doi:10.1080/15548627.2021.1891848)

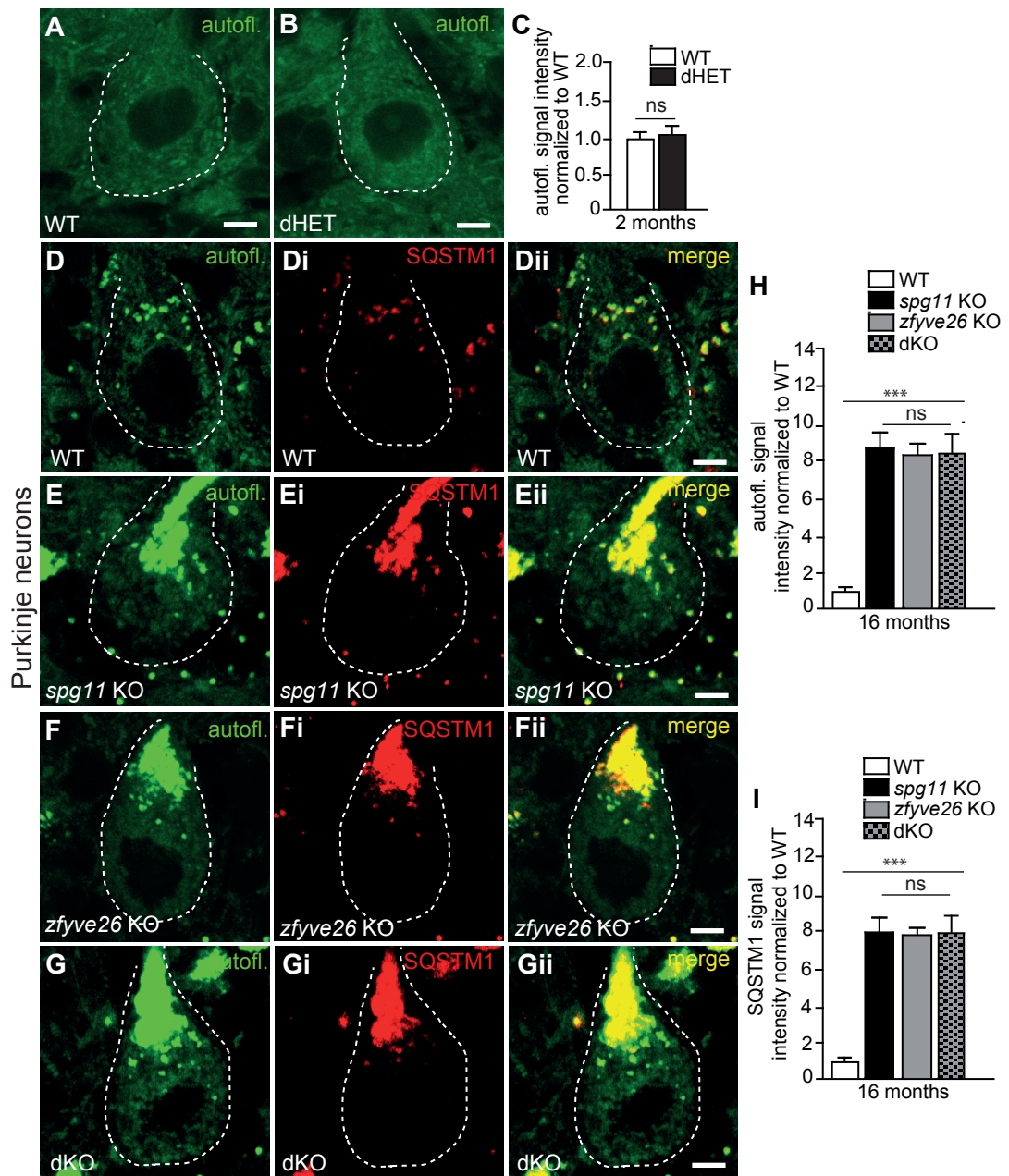

**Figure S1**

Supplement: Supplemental Material [file KAUP_A_1891848_SM4373.zip › Fig. S1.pdf]

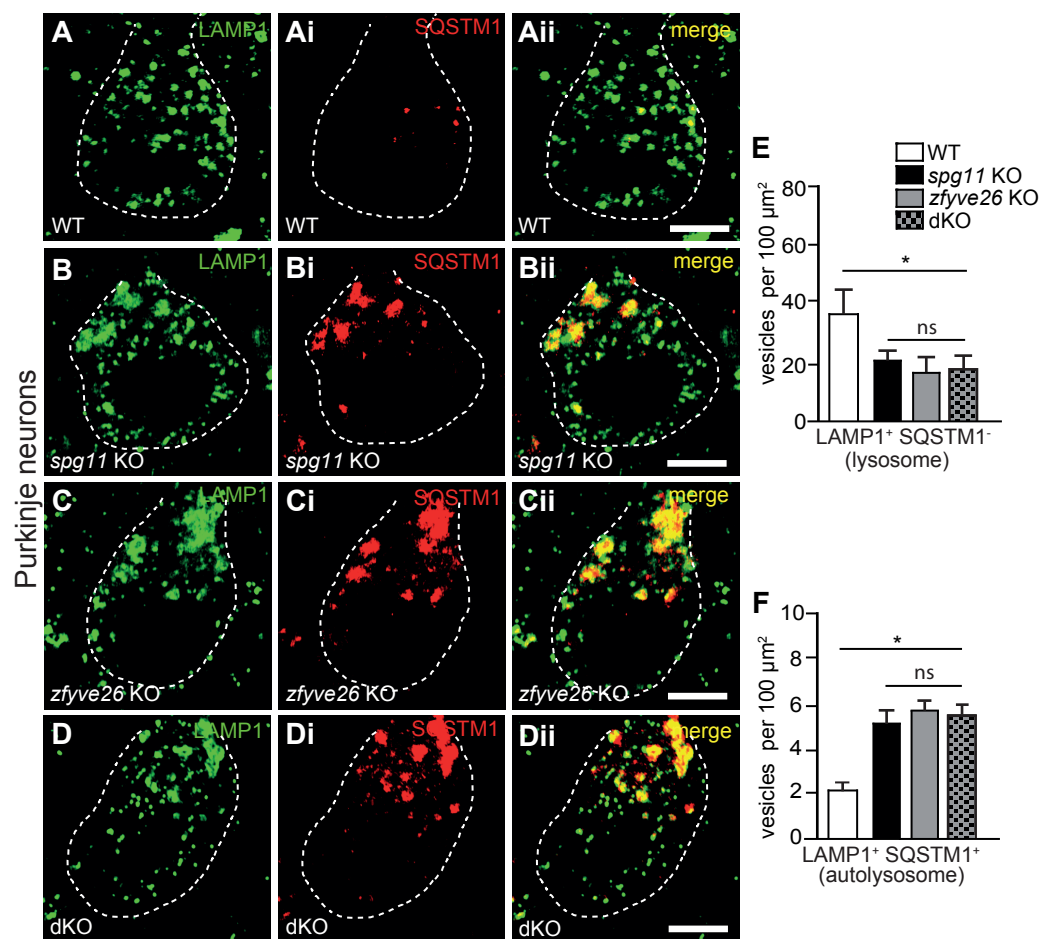

Figure S2

Supplement: Supplemental Material [file KAUP_A_1891848_SM4373.zip › Fig. S2.pdf]

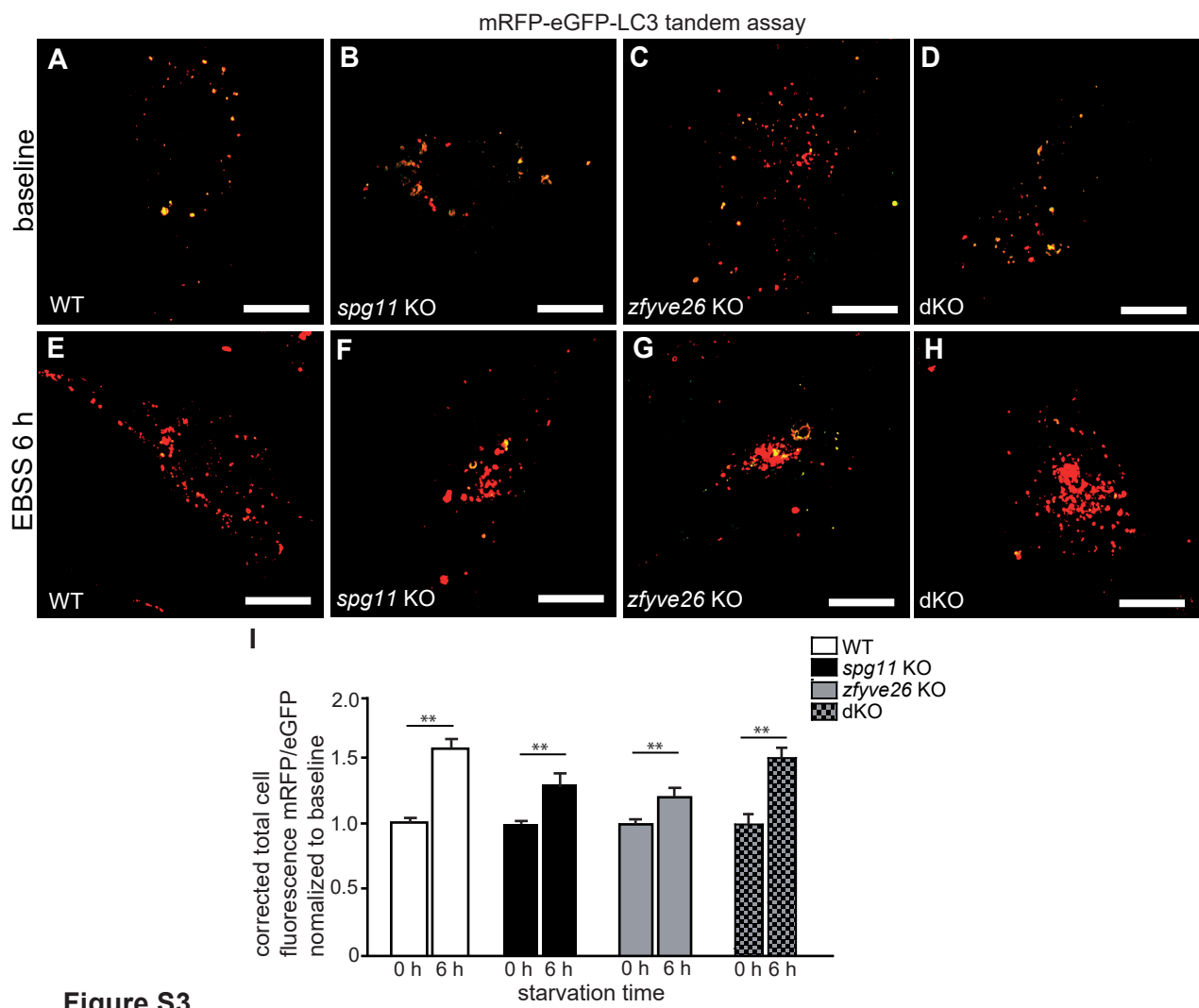

**Figure S3**

Supplement: Supplemental Material [file KAUP_A_1891848_SM4373.zip › Fig. S3.pdf]

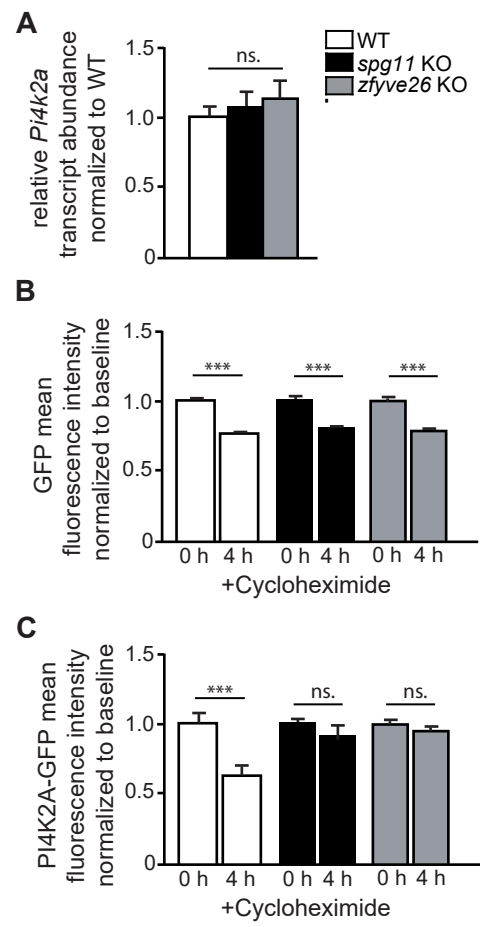

**Figure S4**

Supplement: Supplemental Material [file KAUP_A_1891848_SM4373.zip › Fig. S4.pdf]

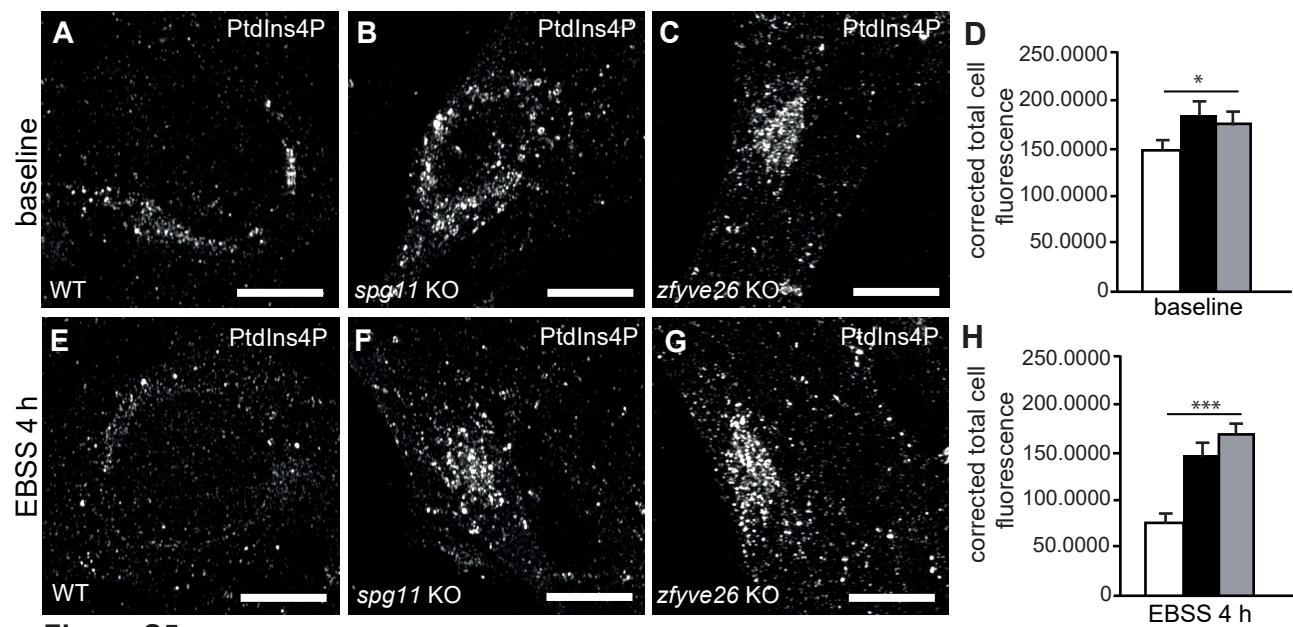

**Figure S5**

Supplement: Supplemental Material [file KAUP_A_1891848_SM4373.zip › Fig. S5.pdf]

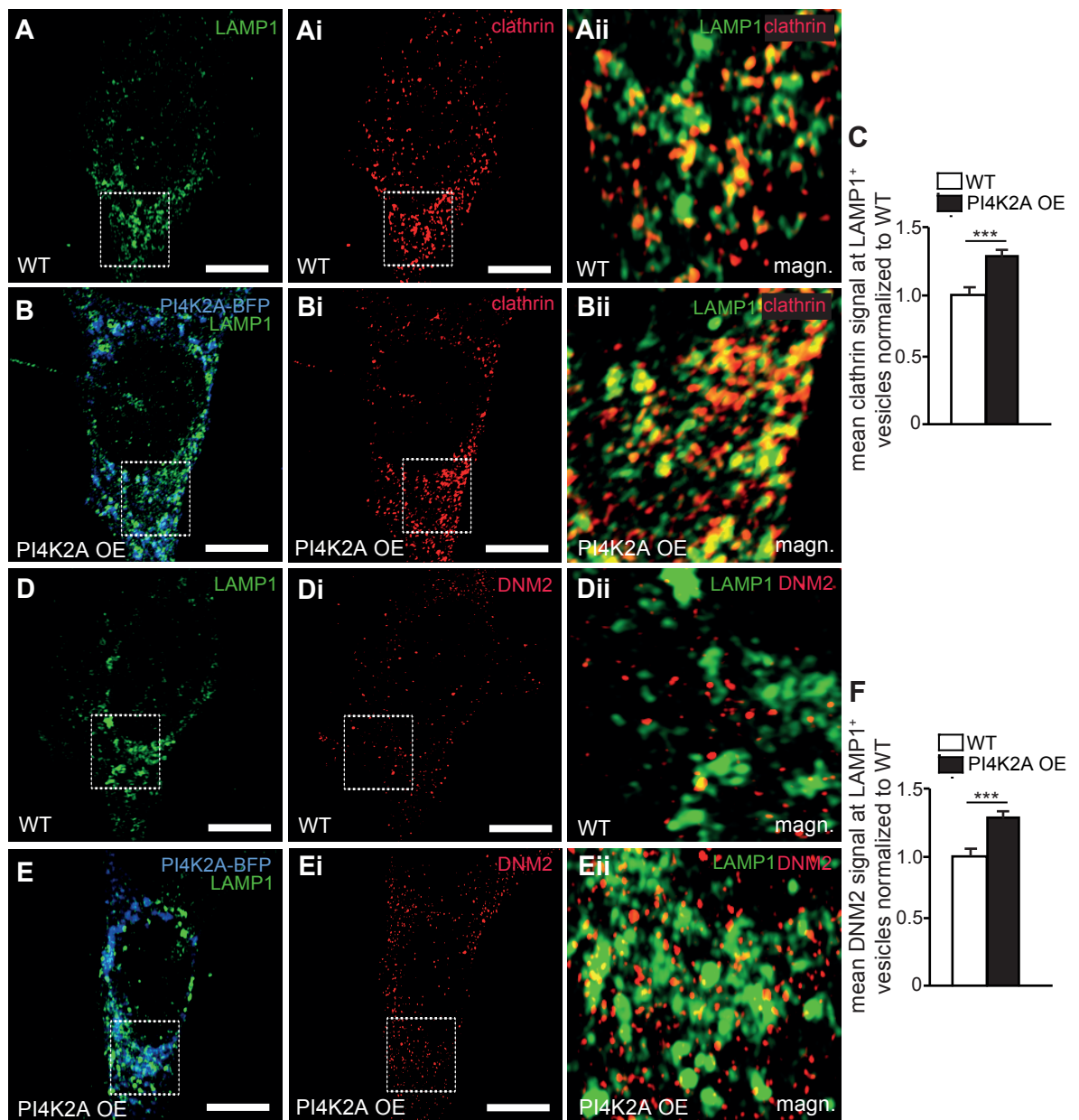

**Figure S6**

Supplement: Supplemental Material [file KAUP_A_1891848_SM4373.zip › Fig. S6.pdf]
